# A Rare Case of Human Diphallia Associated with Hypospadias

**DOI:** 10.1155/2018/8293036

**Published:** 2018-06-13

**Authors:** Andrey Frolov, Yun Tan, Mohammed Waheed-Uz-Zaman Rana, John R. Martin

**Affiliations:** Center for Anatomical Science and Education, Department of Surgery, Saint Louis University School of Medicine, 1402 S. Grand Blvd., Schwitalla Hall M-306, St. Louis, Missouri 63104, USA

## Abstract

Diphallia or penile duplication is a rare congenital variant with an estimated frequency of 1 per 5 to 6 million live births. The extent of duplication varies widely and typically occurs with other malformations including urogenital, gastrointestinal, and musculoskeletal anomalies. Here we present a case of human diphallia that was detected during routine dissection of an 84-year-old cadaver. Upon thorough examination, this case was characterized as a complete bifid penis which was accompanied by hypospadias with no other anatomical abnormalities detected. To gain insights into the etiology of this case, we analyzed DNA procured from the body for putative genetic variants using Next Generation Sequencing (NGS) technology. Our results support clinical observations consistent with human diphallia being a polygenic syndrome and identify new genetic variants that might underlie its etiology.

## 1. Introduction

Diphallia, penile duplication, is a rare condition with an estimated incidence of 1 per 5 to 6 million live births. The first diphallia case was described in 1609 and since then only ~ 100 additional cases have been reported worldwide. The most striking observation in this condition is that each diphallia case is unique and is characterized by a different extent of duplication which is often associated with multiple anomalies including duplication of the bladder, urethra and colon, bladder or cloacal exstrophy, anorectal malformations, and vertebral deformities [1]. Based on the presence of one or two corpora cavernosa in each phallus, diphallia is respectively defined as bifid phallus and true diphallia. The bifid phallus accounts for only one-third of reported diphallia cases [1, 2] which explains the paucity of the data capable of providing insights into its etiology. Embryologically, diphallia is linked to a defect connecting the genital tubercle and urethral folds and seems to occur between the 23rd and 25th days of gestation. It is during this time when strong environmental factors including trauma, drugs, and infection could impact the fetal caudal mass of mesoderm [3], thereby suggesting the involvement of epigenetic mechanism(s) in the development of diphallia. As to the genetic component of diphallia, the respective data, to the best of our knowledge, are limited to one case where the chromosomal balanced reciprocal translocation 46, XY,* t(1,14)(p36.3; q24.3)* was reported and implicated defects in the homeobox genes in diphallia formation [4]. Given the paucity of the information, which could shed light on etiology of diphallia occurrence in humans, we decided to address this shortcoming by performing the respective genetic screen where we probed for single nucleotide variants in the coding (exome) regions of DNA extracted from the archived cadaveric tissue using state-of-the-art NGS technology. The feasibility of the latter approach with regard to the fixed cadaveric tissue has been recently established [5].

## 2. Case Presentation: Anatomical Characterization

**Figure 1:**
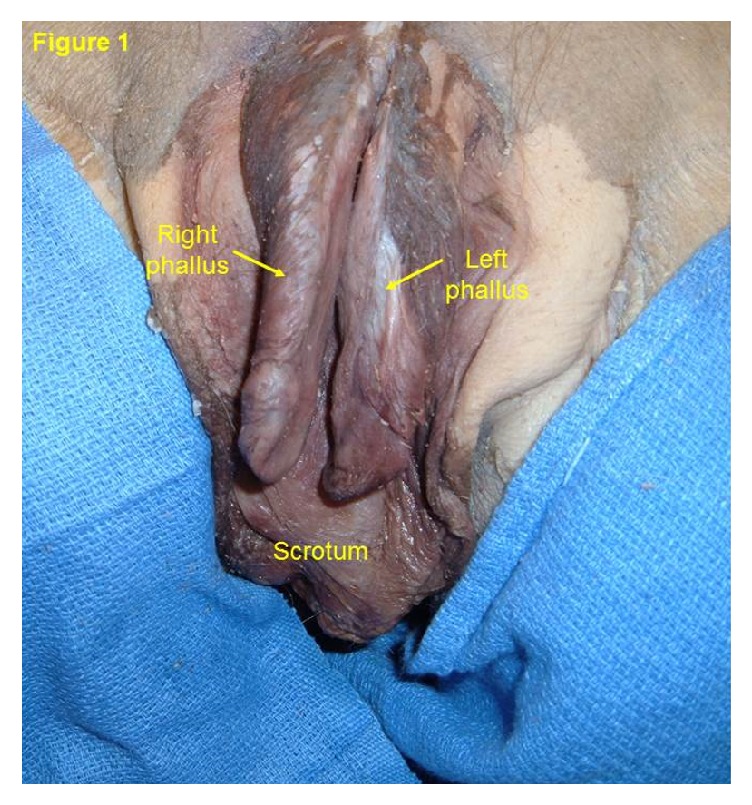
Complete bifid penis. The divided phallus was 9.2 cm long and divided longitudinally into right and left halves. Each half has its own glans and prepuce and no urethra was found in the phallus.

**Figure 2:**
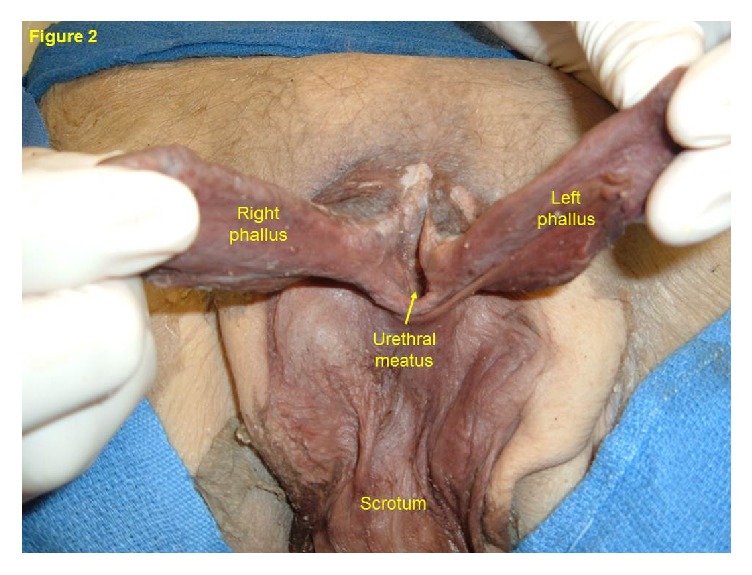
Proximal penoscrotal hypospadias. A urethral meatus opens directly at the base between each half and continues directly into a normally developed urinary bladder.

**Figure 3:**
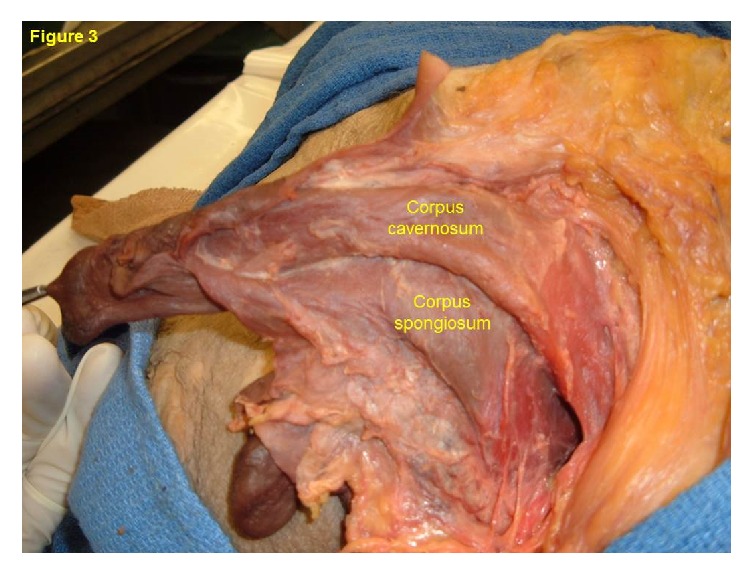
Dissection of the left phallus reveals a corpus cavernosum and corpus spongiosum.

The body of an 84-year-old man was received under the Saint Louis University (SLU) Gift Body Program of the Center for Anatomical Science and Education (CASE) with an informed consent from the donor. Records indicate this man's cause of death was a gastric carcinoma. During routine dissection, a bifid penis was observed. At first glance the phallus resembled that of epispadias, but there were no defects or repair in the external abdominal wall. The pubic hair was sparse and fine. The phallus was 9.2 cm. long, divided longitudinally into right and left parts (Figure 1). Each part had its own glans and prepuce. There was no urethra in the phallus. Rather, a urethral meatus was located at the base of the divided phallus (Figure 2) which is indicative of proximal penoscrotal hypospadias [6, 7]. The urethra continued into the normally developed urinary bladder. The epithelial lining of the urinary bladder extended to 3.9 cm. on the ventral surface of each phallus and histologically it resembled stratified squamous epithelium. The scrotum was large with redundant skin and contained left and right testes, from which extended a normally routed spermatic cord. The right testis was 3.0 long and 2.3 cm wide and the left testis was 1.4 cm long and 0.8 cm wide. The spermatic cord on both sides was 1.2 cm in thickness, continued from each testis and passed through the external and internal inguinal rings and took a normal course to end in well-developed seminal vesicles. The seminal vesicles opened into the urethra. The spermatic cord was of normal thickness and size, contained all of the general coverings, but there was no epididymis. The vas deferens continued directly from each testis to the seminal vesicle of the same side. A small mass, measuring 0.5 cm by 0.5 cm, of hard tissue was found only on the left side and was located at the site of the prostate gland but histologically it did not resemble prostatic glandular tissue. It did not have any connection with the vas deferens or seminal vesicle. Dissection of the phallus revealed a corpus cavernosum and corpus spongiosum in each half of the penis and the corpora spongiosa were well developed as in females (Figure 3). No other abnormalities were observed.

In summary, the anatomical examination of the human cadaver described above characterizes the respective case as a complete bifid penis, a subset of human diphallia, which was accompanied by proximal penoscrotal hypospadias. The lack in this case of additional malformations often associated with diphallia (see above) makes it a perfect model for correct assessment of its etiology.

## 3. Case Presentation: Genetic Analysis

Since human diphallia is an extremely rare anatomical variation as only ~ 100 cases have been reported for over 400 years, we decided to take advantage of this unique opportunity and screen for the diphallia-associated genetic variants to gain important insights into the possible cause(s) of its formation (see Supplementary Materials for experimental details). The advanced bioinformatics analysis of the respective sequencing data identified 476 missense, nonsense, or splicing variants that were annotated and grouped into the following 14 categories:* Development, Morphogenesis, and Tissue Regeneration* (Table S1.1);* Cell Differentiation, Organization, Division, Proliferation, Growth, Migration, Death *(Table S1.2);* Signal Transduction* (Table S1.3);* Hormones and Hormonal Regulation* (Table S1.4);* Gene Transcription and DNA Repair* (Table S1.5);* RNA Transport, Processing and Degradation *(Table S1.6);* Protein Expression, Modification, Transport, Degradation* (Table S1.7);* Membrane Proteins, Receptors, Transporters, and Ion Channels* (Table S1.8);* Immunity and Inflammation* (Table S1.9);* Neuron Differentiation, Migration, and Neurotransmission* (Table S1.10);* Carcinogenesis and Tumor Suppression *(Table S1.11);* Environmental Stress* (Table S1.12);* Miscellaneous Genes* (Table S1.13) and* Uncharacterized or Poorly Characterized Genes *(Table S1.14). Genes with multiple biological functions were placed into each of the respective categories. Tables S1.1-S1.14 can be found in Supplementary Materials. The most relevant and important genetic variants are presented in Table 1 and are discussed below.

## 4. Discussion

The uniqueness and significance of the present case is severalfold.* First, *it can be characterized as a complete bifid penis which accounts for one-third of all reported and extremely rare diphallia cases [1, 2].* Second*, besides hypospadias, no other anomalies often associated with diphallia such as duplication of the bladder, urethra and colon, bladder or cloacal exstrophy, anorectal malformations, and vertebral deformities [1] were observed.* Third,* given the age of the individual's body examined in the current report (84 years old) and the observation that no other major anatomical abnormalities developed through his lifespan, one could conclude that current case of human diphallia associated with hypospadias represents an impairment of developmental process restricted to a specific anatomical area. The latter provides a unique opportunity to gain insights into the respective molecular mechanism(s), thereby assessing correctly the etiology of the external male genitalia duplication and hypospadias in humans.

What are the molecular mechanisms that govern the development of the external male genitalia including those of penis, urethra, and scrotum? In mice, the Sonic hedgehog (Shh) signaling pathway has been identified as a key regulator during initiation of morphologic differentiation of the genital tubercle and its outgrowth, particularly during the formation of urethral tube [8, 9]. In this regard, the differentiation of the genital tubercle after the initiation of Shh signaling from the urethral epithelium leads to the upregulation of bone morphogenetic protein 4 (Bmp4), homeobox protein a13 (Hoxa13), Hoxd13, and Shh receptor, Patc, gene expression. Induction of Bmp4 and Hoxa13/Hoxd13 provides a delicate balance between apoptosis controlled by Bmp4 and proliferation indirectly regulated by Hoxa13/Hoxd13 through the expression of fibroblast growth factor 8 (Fgf8) and wingless-type MMTV (mouse mammary tumor virus) integration site protein 5a (Wnt5a). Such balance is required for the proper differentiation of the genital tubercle and its dysregulation results in hypospadias in which the urethra opens on the underside of the penis and not at the tip. During the later stage of penis development, i.e., the genital tubercle elongation, the spatiotemporal balance between apoptosis and proliferation is maintained by Bmp7 (apoptosis), Fgf8, Fgf10 (proliferation, induced by Shh), and Wnt5a (proliferation) [9]. Therefore, the formation of the external male genitalia in mice represents a tightly regulated developmental process which is governed by a spatiotemporal regulation of the respective genetic program(s).

Our results support clinical observations pointing toward human diphallia being polygenic syndrome and provide several important insights into its etiology.* First*, the respective genetic screen identified mutations in seven genes that could be closely linked to the development of human external genitalia:* BMP4*,* CFAP53*,* DNAH5*,* IFT172, KMT2C, SOX6, and TBX6* (Table 1). Indeed,* CFAP53* (also known as* CCDC11*),* DNAH5*,* IFT172, and TBX6* are involved in the formation and maintenance of cilia, including the primary cilia [10–13], which serves as a platform shared by Shh [14], FGF receptor (FGFR) [15], and Wnt signaling pathways [16, 17]. Therefore, in our case, the spatiotemporal dysregulation of the primary cilia formation and/or function may result in the aberrant SHH-, FGF8/10-, and WNT-dependent cell proliferation signaling that, along with the mutation in* BMP4*, which might negatively affect its proapoptotic function, could disrupt a dynamic equilibrium between cell proliferation and apoptosis [9], thereby skewing the developmental process of human male external genitalia toward diphallia and hypospadias. Because the balance between Shh and Bmp4 signaling was suggested to be a key factor for the developing prostate emanating from the urogenital sinus mesenchyme [9, 18], the absence of the prostate in the current diphallia case (see above) provides strong support to the notion that such balance could be in fact disrupted.

Yet there is also a possibility that primary or additional disruption of the dynamic equilibrium between cell proliferation and apoptosis could come from the mutations in* RYR2 *(BMP signaling),* TTC21B* (ciliary transport, SHH signaling), as well as the genes known to regulate WNT signaling pathway such as ADGRA2,* APC, and CCAR2* (Table 1). The Adgra2 protein serves as a receptor for Wnt7, which is a member of the putative appendicular network for epidermal differentiation during genital tubercle development [19]. Yet the apoptotic arm of the dynamic equilibrium could also be impacted by mutations in* CDK15*,* CYFIP2, DAPK1, DRAM1, MADD, NFKB1, PAK2, TNFAIP8L2, TP53BP2, *and* WWC1 *(Table 1).

*Second,* the mutation in* KMT2C* (Table 1) could indicate the involvement of the epigenetic regulatory mechanism(s) in the above process and support an early hypothesis regarding the detrimental impact of environmental factors on the fetal caudal mass of mesoderm leading to diphallia development [3]. This notion could be further supported by mutations found in* POLR1B *(Table 1), which positively regulates gene transcription through epigenetic mechanism(s) [20] and* HSP90AB1* (Table 1), which modulates activity of several epigenetic regulators [21]. It is tempting to speculate that epigenetic mechanism(s) could also play an important role in the regulation of primary (sensory/signaling) cilium function and the disruption of such mechanism(s) in the spatiotemporal fashion could affect the development of external male genitalia resulting in diphallia and hypospadias.

*Third, *since during prenatal and postnatal periods the development of external genitalia is also influenced by steroid hormones [9, 22–24], the detected mutations in three androgen receptor (AR) coactivators,* NCOA6* (*AIB3/ASC-2*) [25],* TMF1* [25], and* UBE3A* (*E6-AP*) [26] (Table 1), may point toward a novel role of those genes in the hormonal regulation of male sexual development in humans.

*Fourth, *the proximal penoscrotal hypospadias associated with diphallia in the present case could result primarily from* BMP4* mutation (Table 1) although a contribution from* CYP1A1* mutation (Table S1.13) should not be ignored [27].

Finally, the current case has a high educational value because it not only describes a unique andrology syndrome but also provides important insights into its etiology.

## Figures and Tables

**Table 1:** Selected genetic variants associated with present diphallia case.

**Gene**	**Protein Function**
**Development and morphogenesis**	

*BMP4*	Bone morphogenetic protein 4. Cartilage and bone formation. Tooth development, limb formation. Embryonic mammary development. Prostate gland, ureteric bud morphogenesis.

*CFAP53*	Cilia- and flagella-associated protein 53. Beating of primary cilia. Organ laterality during embryogenesis.

*DNAH5*	Dynein heavy chain 5, axonemal. Cilium assembly, cilium movement, determination of left/right asymmetry, heart development.

*IFT172*	Intraflagellar transport protein 172 homolog. Maintenance and formation of cilia. Hedgehog signaling. Dorsal/ventral pattern formation, left/right axis specification, limb, bone, brain development.

*KMT2C*	Histone-lysine N-methyltransferase 2C. Histone methyltransferase. Leukemogenesis and developmental disorder.

*SOX6*	Transcription factor SOX-6. Plays a key role in several developmental processes, including neurogenesis and skeleton formation.

*TBX6*	T-box transcription factor TBX6. Neural development. Morphology and motility of nodal cilia.

*UBE3A*	Ubiquitin-protein ligase E3A. Development. Androgen receptor signaling. Prostate gland growth.

**Signal transduction**	

*ADGRA2*	Adhesion G protein-coupled receptor A2. Functions as a WNT7-specific coactivator of canonical Wnt signaling.

*APC*	Adenomatous polyposis coli protein. Participates in Wnt signaling as a negative regulator.

*CCAR2*	Cell cycle and apoptosis regulator protein 2. Positively regulates the beta-catenin pathway (canonical Wnt signaling pathway) and is required for MCC-mediated repression of the beta-catenin pathway.

*RYR2*	Ryanodine receptor 2. Calcium, BMP signaling.

**Cell differentiation, organization, division, proliferation, growth, migration, death**	

*CDK15*	Cyclin-dependent kinase 15. Anti-apoptotic protein.

*CYFIP2*	Cytoplasmic FMR1-interacting protein 2. T-cell adhesion, apoptosis.

*DAPK1*	Death-associated protein kinase 1. Cell survival, apoptosis, autophagy.

*DRAM1*	DNA damage-regulated autophagy modulator protein 1.

*HSP90AB1*	Heat shock protein HSP 90-beta. Cell cycle control. Epigenetic modifier regulation.

*MADD*	MAP kinase-activating death domain protein. Cell proliferation, survival, death.

*NFKB1*	Nuclear factor NF-kappa-B p105 subunit. Cell growth, differentiation.

*PAK2*	Serine/threonine-protein kinase PAK 2. Cell motility, cell cycle progression, apoptosis or proliferation.

*TNFAIP8L2*	Tumor necrosis factor alpha-induced protein 8-like protein 2. Promotes Fas-induced apoptosis.

*TP53BP2*	Apoptosis-stimulating of p53 protein 2. Regulates apoptosis and cell growth. Impedes cell cycle progression at G2/M.

*WWC1*	Protein KIBRA. Cell proliferation, apoptosis.

**Hormones and hormonal regulation**	

*NCOA6*	Nuclear receptor coactivator 6. Androgen receptor coactivator.

*TMF1*	TATA element modulatory factor. Androgen receptor coactivator.

*UBE3A*	Ubiquitin-protein ligase E3A. Androgen receptor coactivator.

## Data Availability

The datasets and materials used and/or analyzed during the current study are presented in the main paper and additional files.

## Supplementary Materials

Supplementary Materials Supplementary Materials that accompany this paper are Figure S1 and Table S1.
