# Supplementary material for: A Rare Case of Human Diphallia Associated with Hypospadias
Source: Case Rep Urol. 2018 Jun 13;2018:8293036. doi: 10.1155/2018/8293036 (PMC6020512; doi:10.1155/2018/8293036)
Supplement: Supplementary Materials — that accompany this paper are Figure S1 and Table S1. [file 8293036.f1.pdf]

Supplementary materials

A Rare Case of Human Diphallia Associated with Hypospadias

Andrey Frolov, Yun Tan, Mohammed Waheed-Uz-Zaman Rana, and John R. Martin, III*†

Materials and Methods

Human cadaveric body procurement and tissue processing. A donated, male body, received through Saint Louis University (SLU) School of Medicine Gift Body Program, was embalmed through the femoral arteries using a mixture of ethylene glycol and isopropyl alcohol. Dissection of the external genitalia was performed and tissue was extracted, fixed, and paraffin embedded for analysis by Clinical Histopathology Laboratory (SLU School of Medicine) according to standardized procedures. The cadaver used in the current study was obtained from an individual who had given an informed consent to donate his body to the SLU Gift Body Program.

DNA extraction and exome sequencing. The DNA was extracted from the paraffin-embedded right testis specimen using the Omega Bio-tek E.Z.N.A DNA Tissue Kit following the manufacturer's protocol. The concentration of the extracted DNA was 10 ng/µl. Three individual DNA libraries were constructed according to the Illumina Nextera Rapid Exome (62 Mb target region) capture protocol with exome enrichment. The exome sequencing was performed to 30x depth of coverage (~4.5 Gb) on the Illumina HiSeq 2500 NGS platform in the 2x100 base read format. The 30x depth of coverage fulfills a requirement for the detection of human genome mutations (10x to 30x, Illumina). One sequencing run was performed for each of three individual DNA libraries to yield three independent data sets. DNA extraction and exome sequencing were conducted by Omega Bioservices (Norcross, GA).

Bioinformatics analysis. The bioinformatics analysis (the variant calls and functional annotation of genetic variants) was performed by Genome Technology Access Center (GTAC) at Washington University School of Medicine in St. Louis. For that, three independent sequencing data sets (see above) were merged and analyzed as a single file. This was done to minimize the number of variant calls due to duplicate reads, which could arise from the amplification of small DNA fragments (PCR duplicates) that are inevitably present in the DNA extracted from the archived tissue and that could significantly lower the exome coverage thereby reducing the number of detected unique single nucleotide variants. Sequencing reads were aligned to the human reference genome hg190 using NovoAlign (Novocraft Technologies, Petaling Jaya, Malaysia). PCR duplicates were removed from the alignments with Picard MarkDuplicates (Picard) and variants were called using SAMtools (SAMtools). Such an approach yielded ~ 77% reads (coverage) in exome (Supplemental Figure S1) meaning that most of the exome was available for probing. The variants were annotated using SnpSift varType (SnpSift) and ANNOVAR (ANNOVAR) using the following ANNOVAR databases refGene, clinvar_20161128, cosmic70, snp129, snp138, avsnp147, popfreq_all_20150413, exac03, esp6500siv2_all, dbnsfp30a, mcap, revel, dbscsnv11. The resultant data were converted into the Microsoft Excel format and additionally filtered through the following

consecutive steps: *Step 1*: Remove synonymous, nonframeshift deletion, nonframeshift insertion, unknown, and not available (NA) variants; *Step 2*: Remove variants in the ExAc 65,000 exomes with minor allele frequency (MAF) > 0.01; *Step 3*: Keep SIFT-predicted, disease-associated (D) and NA variants; *Step 4*: Keep PolyPhenDiv-predicted D, pathologic (P), and NA variants; *Step 5*: Keep PROVEAN-predicted D and NA variants; *Step 6*: Annotate resultant variants and group them based on their function. The latter was performed by using the UniProtKB Protein knowledgebase as well as by information extracted from the literature obtained through PubMed and Google Scholar database searches.

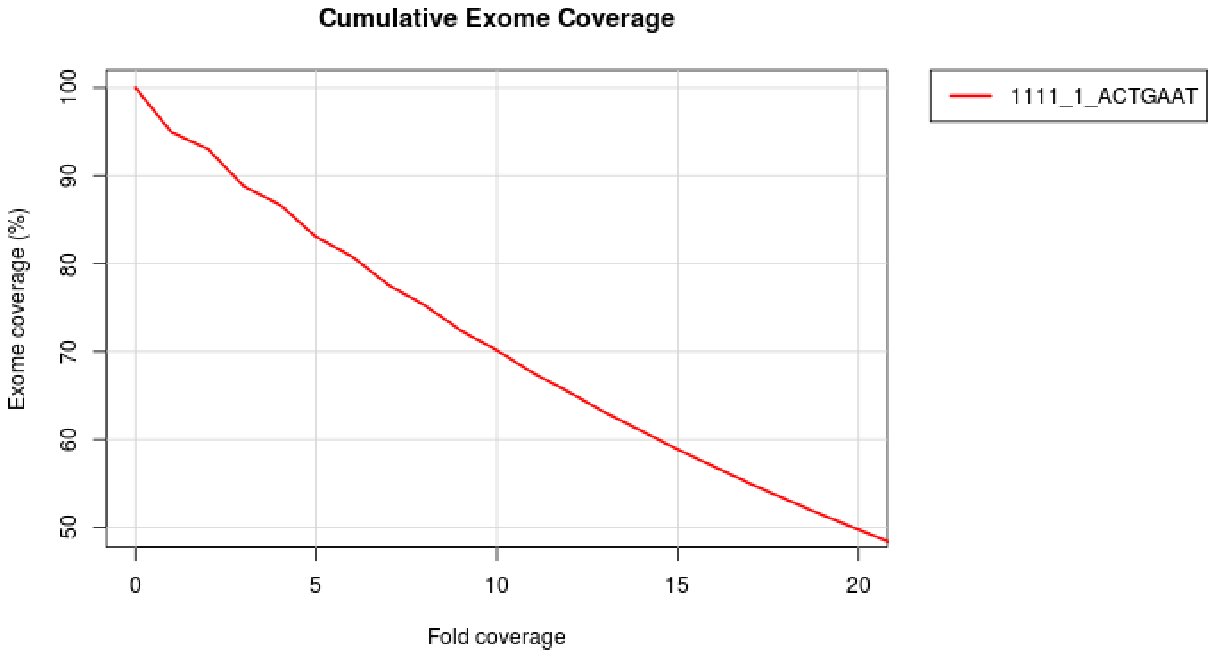

Figure S1. Cumulative exome coverage for NGS sequencing.

Table S1. Complete set of genetic variants associated with present diphallia case.

Table S1.1. Development and morphogenesis.

Gene	Protein Function
ACVR1B	Activin receptor type-1B. Female sexual development. *In utero* embryonic development.
AFG3L2	AFG3-like protein 2. Muscle fiber development.
ARIH2	E3 ubiquitin-protein ligase ARIH2. Myelopoiesis, multicellular organism development.
ATOH8	Protein atonal homolog 8. Early embryonic development.
ATP5A1	ATP synthase subunit alpha, mitochondrial. Embryo development.
BMP4	Bone morphogenetic protein 4. Cartilage and bone formation. Tooth development, limb formation. Embryonic mammary development. Prostate gland, ureteric bud morphogenesis.
CCL13	C-C motif chemokine 13. Organ regeneration.
CFAP53	Cilia- and flagella-associated protein 53. Beating of primary cilia. Organ laterality during embryogenesis.
CHD9	Chromodomain-helicase-DNA-binding protein 9. Osteogenesis.
CNTN5	Contactin-5. Nervous system development.
DEAF1	Deformed epidermal autoregulatory factor 1 homolog. Inhibits cell proliferation. Anatomical structure morphogenesis.
DNAH5	Dynein heavy chain 5, axonemal. Cilium assembly, cilium movement, determination of left/right asymmetry, heart development.
DUOX2	Dual oxidase 2. Thyroid gland development.
FRAS1	Extracellular matrix protein FRAS1. Embryonic limb, metanephros, epithelium morphogeneses.
GAA	Lysosomal alpha-glucosidase. Tissue development.
GRHL1	Grainyhead-like protein 1 homolog. Epithelial development.
IFT172	Intraflagellar transport protein 172 homolog. Maintenance and formation of cilia. Hedgehog signaling. Dorsal/ventral pattern formation, left/right axis specification, limb, bone, brain development.
ITGAX	Integrin alpha-X. Organ morphogenesis.
KDR	Vascular endothelial growth factor receptor 2. Angiogenesis, vascular development, embryonic hematopoiesis.
KMT2C	Histone-lysine N-methyltransferase 2C. Histone methyltransferase. Leukemogenesis and developmental disorder.
MAST2	Microtubule-associated serine/threonine-protein kinase 2. Testis development. (PMID: 25921962).
MEGF8	Multiple epidermal growth factor-like domains protein 8. Digestive tract left/right asymmetry, craniofacial suture morphogenesis, heart left/right asymmetry, embryonic heart tube left/right pattern formation, embryonic heart tube morphogenesis, embryonic limb morphogenesis, embryonic skeletal system morphogenesis.
MSLN	Mesothelin. Pancreas development.
NBEAL2	Neurobeachin-like protein 2. Thrombopoiesis.
NCAN	Neurocan core protein. Skeletal system development.
NFATC4	Nuclear factor of activated T-cells, cytoplasmic 4. Myotube differentiation. Cardiac development.

NHS	Nance-Horan syndrome protein. Eye, tooth, brain, craniofacial development.
NPIPA5	Nuclear pore complex-interacting protein family member A5. Transposition of the great arteries (PMID:26655555).
NRP1	Neuropilin-1. Cardiovascular system development, angiogenesis, organogenesis.
OVGP1	Oviduct-specific glycoprotein. Early embryonic development.
POLR1B	DNA-directed RNA polymerase I subunit RPA2. Embryo implantation and positive regulation of gene expression, epigenetic.
PSMA8	Proteasome subunit alpha type-7-like. Spermatogenesis.
RB1CC1	RB1-inducible coiled-coil protein 1. Muscular differentiation. Fetal hematopoiesis.
RC3H1	Roquin-1. Lymph node development. Spleen development.
SCML4	Sex comb on midleg-like protein 4. Maintains repressive state of homeotic genes throughout development.
SLC26A2	Sulfate transporter. Endochondral bone formation.
SOX6	Transcription factor SOX-6. Plays a key role in several developmental processes, including neurogenesis and skeleton formation.
SPP2	Secreted phosphoprotein 24. Skeletal system development.
TANC2	Protein TANC2. *In utero* embryonic development.
TBX19	T-box transcription factor TBX19. Development. Anatomical structure morphogenesis.
TBX6	T-box transcription factor TBX6. Neural development. Morphology and motility of nodal cilia.
TENM4	Teneurin-4. Neural development.
TEX14	Inactive serine/threonine-protein kinase TEX14. Spermatogenesis, male fertility.
TLL2	Tolloid-like protein 2. Embryonic development, dorsal-ventral patterning. Skeletogenesis.
TRIM45	Tripartite motif-containing protein 45. Bone development.
TRIP12	E3 ubiquitin-protein ligase TRIP12. Embryo development.
UBE3A	Ubiquitin-protein ligase E3A. Development. Androgen receptor signaling. Prostate gland growth.
UBE4B	Ubiquitin conjugation factor E4 B. Myocardium morphogenesis.
VAV3	Guanine nucleotide exchange factor VAV3. Angiogenesis.
WWC1	Protein KIBRA. Restricts proliferation and promotes apoptosis. Negative regulation of organ growth.
ZFP28	Zinc finger protein 28 homolog. Embryonic development.
ZNF7	Zinc finger protein 7. Expressed in human adult testis (PMID:7959769). Development.
ZSCAN31	Zinc finger and SCAN domain-containing protein 31. Development of multiple embryonic organs.

Table S1.2. Signal transduction.

Gene	Protein Function
ACVR1B	Activin receptor type-1B. Transmembrane serine/threonine kinase activin type-1 receptor.
ADGRA2	Adhesion G protein-coupled receptor A2. Functions as a WNT7-specific coactivator of canonical Wnt signaling.
APC	Adenomatous polyposis coli protein. Participates in Wnt signaling as a negative regulator.
ARL13A	ADP-ribosylation factor-like protein 13A. Small GTPase mediated signal transduction.

CCAR2	Cell cycle and apoptosis regulator protein 2. Positively regulates the beta-catenin pathway (canonical Wnt signaling pathway) and is required for MCC-mediated repression of the beta-catenin pathway.
CDK15	Cyclin-dependent kinase 15. Serine/threonine-protein kinase.
CDKL4	Cyclin-dependent kinase-like 4. Serine/threonine kinase activity.
CDKL5	Cyclin-dependent kinase-like 5. Serine/threonine-protein kinase.
CFAP58	Cilia- and flagella-associated protein 58. Fuses with FGFR2 n intra-hepatic cholangio-carcinoma (link).
CMYA5	Cardiomyopathy-associated protein 5. Mediates subcellular compartmentation of protein kinase A via binding to PRKAR2A.
CRHR1	Corticotropin-releasing factor receptor 1. G-protein coupled receptor for corticotropin-releasing factor and urocortin.
CRIPAK	Cysteine-rich PAK1 inhibitor.
CYFIP2	Cytoplasmic FMR1-interacting protein 2. Component of the WAVE1 complex, required for BDNF-NTRK2 endocytic trafficking/ signaling.
DAPK1	Death-associated protein kinase 1. Calcium/calmodulin-dependent serine/threonine kinase.
DLK2	Protein delta homolog 2. Binds calcium. Negatively regulates Notch signaling pathway.
EPS15	Epidermal growth factor receptor substrate 15. Regulates mitogenic signals. Internalization of ligand-inducible receptors of the receptor tyrosine kinase (RTK) type, in particular EGFR.
FARP1	FERM, RhoGEF and pleckstrin domain-containing protein 1. Functions as guanine nucleotide exchange factor for RAC1. Semaphorin signaling.
FGD2	FYVE, RhoGEF and PH domain-containing protein 2. Activates CDC42, a member of the Ras-like family of Rho- and Rac proteins.
GFRA1	GDNF family receptor alpha-1. Mediates GDNF-induced autophosphorylation and activation of RET receptor.
GLG1	Golgi apparatus protein 1. Binds fibroblast growth factor.
GLP2R	Glucagon-like peptide 2 receptor.
GPC1	Glypican-1. Negatively regulates FGFR signaling.
GPR12	G-protein coupled receptor 12. Receptor with constitutive G(s) signaling activity.
GPR153	Probable G-protein coupled receptor 153.
GPR20	G-protein coupled receptor 20. Orphan receptor with constitutive G(i) signaling activity that activate cyclic AMP.
IFT172	Intraflagellar transport protein 172 homolog. Hedgehog signaling.
IGBP1	Immunoglobulin-binding protein 1. Binds to IgM-receptor.
ITGA10	Integrin alpha-10. Integrin-mediated signaling pathway.
ITPR2	Inositol 1,4,5-trisphosphate receptor type 2. Releases intracellular calcium.
KDR	Vascular endothelial growth factor receptor 2. Tyrosine-protein kinase that acts as a cell-surface receptor for VEGFA, VEGFC, VEGFD.
LDLRAD4	Low-density lipoprotein receptor class A domain-containing protein 4. Negative regulator of TGF-beta signaling.
LIPI	Lipase member I. Produces 2-acyl lysophosphatidic acid.
MPZL1	Myelin protein zero-like protein 1. Cell surface receptor. Involved in signal transduction processes.
MST1	Hepatocyte growth factor-like protein. Hepatocyte growth factor receptor signaling pathway.

MYOF	Myoferlin. Implicated in VEGF signal transduction by regulating the levels of the receptor KDR.
NEDD4L	E3 ubiquitin-protein ligase NEDD4-like. Inhibits TGF-beta signaling.
NELL2	Protein kinase C-binding protein NELL2. Modulates MAPK pathways.
NRP1	Neuropilin-1. Bind to various members of the semaphorin family.
OPHN1	Oligophrenin-1. Rho signaling.
PAK2	Serine/threonine-protein kinase PAK 2. Serine/threonine protein kinase. Downstream effector of the small GTPases CDC42 and RAC1.
PLCB1	1-phosphatidylinositol 4,5-bisphosphate phosphodiesterase beta-1
PLCB3	1-phosphatidylinositol 4,5-bisphosphate phosphodiesterase beta-3. Produces second messenger molecules diacylglycerol (DAG) and inositol 1,4,5-trisphosphate (IP3).
PLEKHG4	Puratrophin-1. Intracellular signaling at the Golgi.
PRKAR2B	cAMP-dependent protein kinase type II-beta regulatory subunit. Activates PKA.
PTGIR	Prostacyclin receptor.
RALGAPA2	Ral GTPase-activating protein subunit alpha-2.
RAPGEF3	Rap guanine nucleotide exchange factor 3.
RASAL2	Ras GTPase-activating protein nGAP. Inhibitory regulator of the Ras-cyclic AMP pathway.
RASGRF2	Ras-specific guanine nucleotide-releasing factor 2. Functions as a calcium-regulated nucleotide exchange factor activating both Ras and RAC1.
RNF213	E3 ubiquitin-protein ligase RNF213. Involved in the non-canonical Wnt signaling pathway.
RYR2	Ryanodine receptor 2. Calcium , BMP signaling.
SAMD9L	Sterile alpha motif domain-containing protein 9-like. Mediates down-regulation of growth factor signaling via internalization of growth factor receptors.
STARD8	StAR-related lipid transfer protein 8. Accelerates GTPase activity of RHOA and CDC42, but not RAC1.
STK10	Serine/threonine-protein kinase 10.
SZT2	KICSTOR complex protein SZT2. Negatively regulator of mTORC1 signaling.
TBX6	T-box transcription factor TBX6. Affects Notch signaling through an effect on the morphology and motility of the nodal cilia.
TP53BP2	Apoptosis-stimulating of p53 protein 2. Regulates signal transduction initiated by p53.
TRIO	Triple functional domain protein. Guanine nucleotide exchange factor (GEF) for RHOA and RAC1 GTPases.
TTC21B	Tetratricopeptide repeat protein 21B. Negatively modulates Sonic hedgehog signaling.
UBE3A	Ubiquitin-protein ligase E3A. Androgen receptor signaling pathway.
UHMK1	Serine/threonine-protein kinase Kist.
USP6NL	USP6 N-terminal-like protein. Inhibits EGFR internalization.
VAV3	Guanine nucleotide exchange factor VAV3. Exchange factor for GTP-binding proteins RhoA, RhoG , Rac1. Regulates angiogenesis.
WDFY2	WD repeat and FYVE domain-containing protein 2. Mediates interaction between PRKCZ and its substrate VAMP2. Controls AKT2 signaling.
WNK3	Serine/threonine-protein kinase WNK3.
WWC1	Protein KIBRA. Probable regulator of the Hippo/SWH (Sav/Wts/Hpo) signaling pathway.
ZNF7	Zinc finger protein 7. Regulates non-canonical NF-kB pathway (PMID:24008839).

Table S1.3. Cell differentiation, organization, division, proliferation, growth, migration, death.

Gene	Protein Function
ARIH2	E3 ubiquitin-protein ligase ARIH2. Cell growth.
ATG2A	Autophagy-related protein 2 homolog A.
ATOH8	Protein atonal homolog 8. Myoblast differentiation phase.
BCS1L	Mitochondrial chaperone BCS1. Assembly of mitochondrial respiratory chain complex III.
CACNA1D	Voltage-dependent L-type calcium channel subunit alpha-1D. Cell motility, division, death.
CADM2	Cell adhesion molecule 2. Adhesion molecule.
CAMSAP1	Calmodulin-regulated spectrin-associated protein 1. Cell morphology/cytoskeletal organization.
CAPN1	Calpain-1 catalytic subunit. Cytoskeletal remodeling.
CASZ1	Zinc finger protein castor homolog 1. Neuron differentiation.
CCAR2	Cell cycle and apoptosis regulator protein 2.
CCDC65	Coiled-coil domain-containing protein 65. Motile cilia function.
CCT8	T-complex protein 1 subunit theta. Ciliogenesis.
CDC27	Cell division cycle protein 27 homolog. Mitosis.
CDH6	Cadherin-6. Cell sorting.
CDK15	Cyclin-dependent kinase 15. Antiapoptotic protein.
CENPM	Centromere protein M. Mitosis.
CEP164	Centrosomal protein of 164 kDa. Microtubule organization and/or maintenance.
CEP68	Centrosomal protein of 68 kDa.
CFAP53	Cilia- and flagella-associated protein 53. Primary cilia motility.
CFAP57	Cilia- and flagella-associated protein 57.
CFAP58	Cilia- and flagella-associated protein 58.
CHD9	Chromodomain-helicase-DNA-binding protein 9. Cell division /differentiation.
CLASP2	CLIP-associating protein 2. Microtubule plus-end tracking protein.
CMYA5	Cardiomyopathy-associated protein 5. Negative regulation of skeletal muscle regeneration.
COL4A6	Collagen alpha-6(IV) chain. Cell maintenance.
CYFIP2	Cytoplasmic FMR1-interacting protein 2. T-cell adhesion/apoptosis.
DAPK1	Death-associated protein kinase 1. Cell survival, apoptosis, autophagy.
DGCR8	Microprocessor complex subunit DGCR8. Silencing of embryonic stem cell self-renewal.
DLK2	Protein delta homolog 2. Adipocyte differentiation.
DNAH10	Dynein heavy chain 10, axonemal. Force generating protein of respiratory cilia.
DNAH11	Dynein heavy chain 11, axonemal. Force generating protein of respiratory cilia.
DNAH2	Dynein heavy chain 2, axonemal. Force generating protein of respiratory cilia.
DNAH3	Dynein heavy chain 3, axonemal. Force generating protein of respiratory cilia.
DNAH5	Dynein heavy chain 5, axonemal. Cilium assembly/movement.
DNAH7	Dynein heavy chain 7, axonemal. Force generating protein of respiratory cilia.
DNAH9	Dynein heavy chain 9, axonemal. Force generating protein of respiratory cilia.
DNASE1L3	Deoxyribonuclease gamma. Apoptosis/necrosis.
DRAM1	DNA damage-regulated autophagy modulator protein 1.
DYSF	Dysferlin. Plasma membrane resealing.

ENAH	Protein enabled homolog. Cell migration.
EPB41L5	Band 4.1-like protein 5. Epithelial cell polarity.
EPS15	Epidermal growth factor receptor substrate 15. Cell growth regulation, cell proliferation.
FAT1	Protocadherin Fat 1. Cell polarization and migration.
FGD2	FYVE, RhoGEF and PH domain-containing protein 2. Positively regulates apoptotic process and cell shape.
FLNC	Filamin-C. Myogenesis. Structural integrity of muscle fibers.
FNBP1L	Formin-binding protein 1-like. Required to coordinate membrane tubulation with reorganization of the actin cytoskeleton during endocytosis.
HAUS5	HAUS augmin-like complex subunit 5. Cytokinesis.
HOOK2	Protein Hook homolog 2. Vesicular trafficking.
HPS3	Hermansky-Pudlak syndrome 3 protein. Melanosome biogenesis.
HSP90AB1	Heat shock protein HSP 90-beta. Cell cycle control. Epigenetic modifier regulation.
IGSF10	Immunoglobulin superfamily member 10. Maintenance of osteochondroprogenitor cells.
ITGA10	Integrin alpha-10. Cell-matrix adhesion.
ITGAD	Integrin alpha-D. Phagocytosis.
ITGAX	Integrin alpha-X. Monocyte adhesion and chemotaxis.
KDR	Vascular endothelial growth factor receptor 2. Cell proliferation, survival, migration, differentiation.
KHDRBS3	KH domain-containing, RNA-binding, signal transduction-associated protein 3. Negative regulation of cell growth/proliferation.
KIF21B	Kinesin-like protein KIF21B. Plus-end directed microtubule-dependent motor protein.
KNCN	Kinocilin. Stabilizes dense microtubular networks.
LRRC45	Leucine-rich repeat-containing protein 45. Component of the proteinaceous fiber-like linker between two centrioles. Required for centrosome cohesion.
MADD	MAP kinase-activating death domain protein. Cell proliferation, survival, death.
MICAL3	[F-actin]-methionine sulfoxide oxidase MICAL3. Cytoskeleton.
MYO5A	Unconventional myosin-Va. Intracellular trafficking.
MYOF	Myoferlin. Plasmalemma repair mechanism of endothelial cells.
NCAPD2	Condensin complex subunit 1. Mitosis.
NFATC4	Nuclear factor of activated T-cells, cytoplasmic 4. Adipocyte and myotube differentiation.
NFKB1	Nuclear factor NF-kappa-B p105 subunit. Cell growth/differentiation.
NHS	Nance-Horan syndrome protein. Cell motility.
NHSL2	NHS-like protein 2. Cell differentiation.
NR3C1	Glucocorticoid receptor. Cell proliferation/differentiation.
NRP1	Neuropilin-1. Angiogenesis.
NTM	Neurotrimin. Cell adhesion.
OSGIN1	Oxidative stress-induced growth inhibitor 1. Cell differentiation, proliferation, death.
PAK2	Serine/threonine-protein kinase PAK 2. Cell motility, cell cycle progression, apoptosis or proliferation.
PARD3	Partitioning defective 3 homolog. Asymmetrical cell division/polarization.
PCDH7	Protocadherin-7. Cell adhesion. Platelet degranulation.
PLEKHG4	Puratrophin-1. Cytoskeleton dynamics at the Golgi.

RB1CC1	RB1-inducible coiled-coil protein 1. Autophagy, muscular differentiation, neuronal homeostasis.
RNASE3	Eosinophil cationic protein. Cytotoxin and helminthotoxin.
RPRD1A	Regulation of nuclear pre-mRNA domain-containing protein 1A. Cell cycle regulation.
SENP6	Sentrin-specific protease 6. Kinetochore and spindle assembly.
SKA3	Spindle and kinetochore-associated protein 3. Cell division.
SLAIN1	SLAIN motif-containing protein 1. Embryonic stem cells.
SPEG	Striated muscle preferentially expressed protein kinase. Cell growth/differentiation.
SVIL	Supervillin. Myogenesis.
TBX19	T-box transcription factor TBX19. Cell differentiation/proliferation.
TBX6	T-box transcription factor TBX6. Cell differentiation.
TCHP	Trichoplein keratin filament-binding protein. Pro-apoptotic. Inhibits cell growth.
TEP1	Telomerase protein component 1. Cell division.
TEX14	Inactive serine/threonine-protein kinase TEX14. Meiosis/mitosis.
TGM1	Protein-glutamine gamma-glutamyltransferase K. Positively regulates cell cycle.
TJAP1	Tight junction-associated protein 1. Golgi organization.
TNFAIP8L2	Tumor necrosis factor alpha-induced protein 8-like protein 2. Promotes Fas-induced apoptosis.
TP53BP2	Apoptosis-stimulating of p53 protein 2. Regulates apoptosis and cell growth. Impedes cell cycle progression at G2/M.
TPP2	Tripeptidyl-peptidase 2. Stimulates adipogenesis.
TRIO	Triple functional domain protein. Cell migration/growth.
TTC21B	Tetratricopeptide repeat protein 21B. Retrograde ciliary transport.
TUBGCP2	Gamma-tubulin complex component 2. Mitosis.
TUBGCP6	Gamma-tubulin complex component 6. Mitosis.
UBE3A	Ubiquitin-protein ligase E3A. Circadian clock regulation.
UHMK1	Serine/threonine-protein kinase Kist. Controls cell cycle progression in G1 phase.
UHRF1BP1	UHRF1-binding protein 1. Cell growth.
ULK4	Serine/threonine-protein kinase ULK4. Cell motility.
USP36	Ubiquitin carboxyl-terminal hydrolase 36. Stem cell maintenance.
WNK3	Serine/threonine-protein kinase WNK3. Cell survival/proliferation.
WWC1	Protein KIBRA. Cell proliferation/apoptosis.
ZMYM3	Zinc finger MYM-type protein 3. Cell maintenance.

Table S1.4. Hormones and hormonal regulation.

Gene	Protein Function
CRHR1	Corticotropin-releasing factor receptor 1. Hormone signaling /secretion.
CRIPAK	Cysteine-rich PAK1 inhibitor. Hormonal independence.
CYP11B2	Cytochrome P450 11B2, mitochondrial. Hormone metabolism.
CYP21A2	Steroid 21-hydroxylase. Hormone biosynthesis.
DUOX1	Dual oxidase 1. Hormone biosynthesis.
DUOX2	Dual oxidase 2. Hormone biosynthesis.

FDXR	NADPH:adrenodoxin oxidoreductase, mitochondrial. Hormone biosynthesis, metabolism.
MVD	Diphosphomevalonate decarboxylase. Hormone biosynthesis.
NCOA6	Nuclear receptor coactivator 6. Androgen receptor coactivator.
NELL2	Protein kinase C-binding protein NELL2. Hormone secretion.
NR3C1	Glucocorticoid receptor.
SERPINA12	Serpin A12. Modulates insulin action.
TMF1	TATA element modulatory factor. Androgen receptor coactivator.
UBE3A	Ubiquitin-protein ligase E3A. Androgen receptor coactivator.

Table S1.5. Gene transcription and DNA repair.

Gene	Protein Function
ATAD2	ATPase family AAA domain-containing protein 2. May be a transcriptional coactivator of the nuclear receptor ESR1 required to induce the expression of a subset of estradiol target genes, such as CCND1, MYC and E2F1. May be required for histone hyperacetylation. May regulate chromatin organization and negatively regulate chromatin silencing.
ATOH8	Protein atonal homolog 8. Transcription factor that binds a palindromic (canonical) core consensus DNA sequence 5'-CANNTG- 3' known as an E-box element, possibly as a heterodimer with other bHLH proteins (PubMed:24236640). Regulates endothelial cell proliferation , migration and tube-like structures formation (PubMed:24463812). During early embryonic development is involved in tissue-specific differentiation processes that are dependent on class II bHLH factors and namely modulates the differentiation program initiated by the pro-endocrine factor NEUROG3 (By similarity). During myogenesis, may play a role during the transition of myoblasts from the proliferative phase to the differentiation phase (By similarity). Tube formation.
BRPF3	Bromodomain and PHD finger-containing protein 3. Component of the MOZ/MORF complex which has a histone H3 acetyltransferase activity.
CACNA1D	Voltage-dependent L-type calcium channel subunit alpha-1D. Voltage-sensitive calcium channels (VSCC) mediate the entry of calcium ions into excitable cells and are also involved in a variety of calcium-dependent processes, including muscle contraction, hormone or neurotransmitter release, gene expression, cell motility, cell division and cell death.
CAMTA1	Calmodulin-binding transcription activator 1. Transcriptional activator. May act as a tumor suppressor.
CASZ1	Zinc finger protein castor homolog 1. Transcriptional activator (PubMed:23639441, PubMed:27693370). Involved in vascular assembly and morphogenesis through direct transcriptional regulation of EGFL7 (PubMed:23639441). May also regulate neuron differentiation.

CCAR2	Cell cycle and apoptosis regulator protein 2. Core component of the DBIRD complex, a multiprotein complex that acts at the interface between core mRNP particles and RNA polymerase II (RNAPII) and integrates transcript elongation with the regulation of alternative splicing: the DBIRD complex affects local transcript elongation rates and alternative splicing of a large set of exons embedded in (A + T)-rich DNA regions. Inhibits SIRT1 deacetylase activity leading to increasing levels of p53/TP53 acetylation and p53-mediated apoptosis. Inhibits SUV39H1 methyltransferase activity. As part of a histone H3-specific methyltransferase complex may mediate ligand-dependent transcriptional activation by nuclear hormone receptors. Positively regulates the beta-catenin pathway (canonical Wnt signaling pathway) and is required for MCC-mediated repression of the beta-catenin pathway (PubMed:24824780).
CHD9	Chromodomain-helicase-DNA-binding protein 9. Acts as a transcriptional coactivator for PPARA and possibly other nuclear receptors. Proposed to be a ATP-dependent chromatin remodeling protein. Has DNA-dependent ATPase activity and binds to A/T-rich DNA. Associates with A/T-rich regulatory regions in promoters of genes that participate in the differentiation of progenitors during osteogenesis (By similarity). Regulates covalent chromatin modification.
CMYA5	Cardiomyopathy-associated protein 5. May serve as an anchoring protein that mediates the subcellular compartmentation of protein kinase A (PKA) via binding to PRKAR2A (By similarity). May function as a repressor of calcineurin-mediated transcriptional activity. May attenuate calcineurin ability to induce slow-fiber gene program in muscle and may negatively modulate skeletal muscle regeneration (By similarity).
CRIPAK	Cysteine-rich PAK1 inhibitor. Negative regulator of PAK1. It has been suggested that the loss of CRIPAK in breast tumors might contribute to hormonal independence.
DEAF1	Deformed epidermal autoregulatory factor 1 homolog. Transcription factor that binds to sequence with multiple copies of 5'-TTC[CG]G-3' present in its own promoter and that of the HNRPA2B1 gene. Down-regulates transcription of these genes. Binds to the retinoic acid response element (RARE) 5'-AGGGTTCACCGAAAGTTCA-3'. Activates the proenkephalin gene independently of promoter binding, probably through protein-protein interaction. When secreted, behaves as an inhibitor of cell proliferation, by arresting cells in the G0 or G1 phase. Required for neural tube closure and skeletal patterning. Regulates epithelial cell proliferation and side-branching in the mammary gland. Defective DEAF1 could confer a growth advantage to the mutated cells influencing the development and progression of neoplasia, e.g. in the case of colorectal carcinomas. May regulate anatomical structure morphogenesis.
DHX38	Pre-mRNA-splicing factor ATP-dependent RNA helicase PRP16.
DNMT3B	DNA (cytosine-5)-methyltransferase 3B. Required for genome-wide de novo methylation and is essential for the establishment of DNA methylation patterns during development. DNA methylation is coordinated with methylation of histones. May preferentially methylates nucleosomal DNA within the nucleosome core region. May function as transcriptional co-repressor by associating with CBX4 and independently of DNA methylation. Seems to be involved in gene silencing (By similarity).
EID3	EP300-interacting inhibitor of differentiation 3. Tissue-specific component of the SMC5-SMC6 complex, a complex involved in repair of DNA double-strand breaks by homologous recombination.

EXOSC10	Exosome component 10. Putative catalytic component of the RNA exosome complex which has 3'->5' exoribonuclease activity and participates in a multitude of cellular RNA processing and degradation events. May be involved in dosage compensation by inactivation of X chromosome.
GABPB2	GA-binding protein subunit beta-2. May function as transcription factor capable of interacting with purine rich repeats (GA repeats).
GRHL1	Grainyhead-like protein 1 homolog. Transcription factor involved in epithelial development. GRHL1, GRHL2, and GRHL3 have related but remarkably different functions during embryogenesis because of their differential spatiotemporal expression patterns during development.
HIRA	Protein HIRA. Cooperates with ASF1A to promote replication-independent chromatin assembly. Required for the periodic repression of histone gene transcription during the cell cycle.
HNRNPUL1	Heterogeneous nuclear ribonucleoprotein U-like protein 1. Acts as a basic transcriptional regulator. Represses basic transcription driven by several virus and cellular promoters. When associated with BRD7, activates transcription of glucocorticoid- responsive promoter in the absence of ligand-stimulation.
HSP90AB1	Heat shock protein HSP 90-beta. Molecular chaperone that promotes the maturation, structural maintenance and proper regulation of specific target proteins involved for instance in cell cycle control and signal transduction. Apart from its chaperone activity, it also plays a role in the regulation of the transcription machinery. HSP90 and its co-chaperones modulate transcription at least at three different levels. In the first place, they alter the steady-state levels of certain transcription factors in response to various physiological cues. Second, they modulate the activity of certain epigenetic modifiers, such as histone deacetylases or DNA methyl transferases, and thereby respond to the change in the environment. Third, they participate in the eviction of histones from the promoter region of certain genes and thereby turn on gene expression (PubMed:25973397).
IKZF2	Zinc finger protein Helios. Involved in both negative and positive regulation of transcription from RNA polymerase II promoter.
INTS9	Integrator complex subunit 9. Component of the Integrator (INT) complex, a complex involved in the small nuclear RNAs (snRNA) U1 and U2 transcription and in their 3'-box-dependent processing. Binds zinc.
KMT2C	Histone-lysine N-methyltransferase 2C. Histone methyltransferase. Methylates 'Lys-4' of histone H3. H3 'Lys-4' methylation represents a specific tag for epigenetic transcriptional activation. May be involved in leukemogenesis and developmental disorder.
LIG3	DNA ligase 3. Isoform 3 functions as heterodimer with DNA-repair protein XRCC1 in the nucleus and can correct defective DNA strand-break repair and sister chromatid exchange following treatment with ionizing radiation and alkylating agents.
MIER2	Mesoderm induction early response protein 2. Transcriptional repressor.
NCOA6	Nuclear receptor coactivator 6. Nuclear receptor coactivator that directly binds nuclear receptors and stimulates the transcriptional activities in a hormone-dependent fashion. Coactivates expression in an agonist- and AF2-dependent manner. Involved in the coactivation of different nuclear receptors, such as for steroids (GR and ERs), retinoids (RARs and RXRs), thyroid hormone (TRs), vitamin D3 (VDR) and prostanoids (PPARs).
NCOR2	Nuclear receptor corepressor 2. Transcriptional corepressor. Mediates the transcriptional repression activity of some nuclear receptors by promoting chromatin condensation, thus preventing access of the basal transcription.

NFATC2IP	NFATC2-interacting protein. In T-helper 2 (Th2) cells, regulates the magnitude of NFAT-driven transcription of a specific subset of cytokine genes, including IL3, IL4, IL5 and IL13, but not IL2. Recruits PRMT1 to the IL4 promoter; this leads to enhancement of histone H4 'Arg-3'-methylation and facilitates subsequent histone acetylation at the IL4 locus, thus promotes robust cytokine expression (By similarity).
NFKB1	Nuclear factor NF-kappa-B p105 subunit. NF-kappa-B is a pleiotropic transcription factor present in almost all cell types and is the endpoint of a series of signal transduction events that are initiated by a vast array of stimuli related to many biological processes such as inflammation, immunity, differentiation, cell growth, tumorigenesis and apoptosis. Also involved in regulation of transcription from RNA polymerase II promoter.
NFX1	Transcriptional repressor NF-X1. Binds to the X-box motif of MHC class II genes and represses their expression. May have transcription factor activity.
NR1I2	Nuclear receptor subfamily 1 group I member 2. Nuclear receptor that binds and is activated by variety of endogenous and xenobiotic compounds. Transcription factor that activates the transcription of multiple genes involved in the metabolism and secretion of potentially harmful xenobiotics, drugs and endogenous compounds.
NRIP2	Nuclear receptor-interacting protein 2. Down-regulates transcriptional activation by nuclear receptors such as NR1F2.
NT5C2	Cytosolic purine 5'-nucleotidase. May have a critical role in the maintenance of a constant composition of intracellular purine/pyrimidine nucleotides in cooperation with other nucleotidases.
PAK2	Serine/threonine-protein kinase PAK 2. Serine/threonine protein kinase that plays a role in a variety of different signaling pathways including cytoskeleton regulation, cell motility, cell cycle progression, apoptosis or proliferation. Acts as downstream effector of the small GTPases CDC42 and RAC1. Phosphorylates many other substrates including histone H4 to promote assembly of H3.3 and H4 into nucleosomes.
PALB2	Partner and localizer of BRCA2. Plays a critical role in homologous recombination repair (HRR) through its ability to recruit BRCA2 and RAD51 to DNA breaks.
PARP2	Poly [ADP-ribose] polymerase 2. Involved in the base excision repair (BER) pathway, by catalyzing the poly(ADP-ribosylation of a limited number of acceptor proteins involved in chromatin architecture and in DNA metabolism (PubMed:10364231, PubMed:28190768). This modification follows DNA damages and appears as an obligatory step in a detection /signaling pathway leading to the reparation of DNA strand breaks (PubMed:10364231).
PKM	Pyruvate kinase PKM. Glycolytic enzyme that catalyzes the transfer of a phosphoryl group from phosphoenolpyruvate (PEP) to ADP, generating ATP. Stimulates POU5F1-mediated transcriptional activation. Plays a general role in caspase independent cell death of tumor cells. May regulate animal organ regeneration.
POLR1B	DNA-directed RNA polymerase I subunit RPA2. DNA-dependent RNA polymerase catalyzes the transcription of DNA into RNA using the four ribonucleoside triphosphates as substrates. Second largest core component of RNA polymerase I which synthesizes ribosomal RNA precursors. Involved in regulation of embryo implantation and positive regulation of gene expression, epigenetic.
PSKH1	Serine/threonine-protein kinase H1. May be a SFC-associated serine kinase (splicing factor compartment-associated serine kinase) with a role in intranuclear SR protein (non-snRNP splicing factors containing a serine/arginine-rich domain) trafficking and pre-mRNA processing.
RAD50	DNA repair protein RAD50. Component of the MRN complex, which plays a central role in

	double-strand break (DSB) repair, DNA recombination, maintenance of telomere integrity and meiosis.
SCAF4	Splicing factor, arginine/serine-rich 15. May act to physically and functionally link transcription and pre-mRNA processing.
SCML4	Sex comb on midleg-like protein 4. Putative Polycomb group (PcG) protein. PcG proteins act by forming multiprotein complexes, which are required to maintain the transcriptionally repressive state of homeotic genes throughout development (By similarity).
SOX6	Transcription factor SOX-6. Transcriptional activator. Binds specifically to the DNA sequence 5'-AACAAT-3'. Plays a key role in several developmental processes, including neurogenesis and skeleton formation.
TBX19	T-box transcription factor TBX19. Transcriptional regulator involved in developmental processes. Can activate POMC gene expression and repress the alpha glycoprotein subunit and thyroid-stimulating hormone beta promoters. Anatomical structure morphogenesis. Pituitary gland development. Cell fate commitment. Regulation of cell differentiation and proliferation.
TDRD3	Tudor domain-containing protein 3. Scaffolding protein that specifically recognizes and binds dimethylarginine-containing proteins. In nucleus, acts as a coactivator: recognizes and binds asymmetric dimethylation on the core histone tails associated with transcriptional activation (H3R17me2a and H4R3me2a) and recruits proteins at these arginine-methylated loci. In cytoplasm, may play a role in the assembly and/or disassembly of mRNA stress granules and in the regulation of translation of target mRNAs by binding Arg/Gly-rich motifs (GAR) in dimethylarginine-containing proteins. Transcriptional coactivator.
TNRC6C	Trinucleotide repeat-containing gene 6C protein. Plays a role in RNA-mediated gene silencing by micro-RNAs (miRNAs). Required for miRNA-dependent translational repression of complementary mRNAs by argonaute family proteins.
TRIP12	E3 ubiquitin-protein ligase TRIP12. E3 ubiquitin-protein ligase involved in ubiquitin fusion degradation (UFD) pathway and regulation of DNA repair. Acts as a key regulator of DNA damage response by acting as a suppressor of RNF168, an E3 ubiquitin-protein ligase that promotes accumulation of 'Lys-63'-linked histone H2A and H2AX at DNA damage sites, thereby acting as a guard against excessive spreading of ubiquitinated chromatin at damaged chromosomes. Involved in regulation of embryo development and negative regulation of histone H2A K63-linked ubiquitination.
TSC22D1	TSC22 domain family protein 1. Transcriptional repressor. Acts on the C-type natriuretic peptide (CNP) promoter.
USP36	Ubiquitin carboxyl-terminal hydrolase 36. May be required for maintaining multiple types of adult stem cells. May function as a transcriptional repressor by continually deubiquiting histone H2B at the promoters of genes critical for cellular differentiation, thereby preventing histone H3 'Lys-4' trimethylation (H3K4).
UTP4	U3 small nucleolar RNA-associated protein 4 homolog. Ribosome biogenesis factor. Involved in nucleolar processing of pre-18S ribosomal RNA. Involved in small subunit (SSU) pre-rRNA processing at sites A', A0, 1 and 2b.
ZFP28	Zinc finger protein 28 homolog. May be involved in transcriptional regulation. May have a role in embryonic development.
ZMYM3	Zinc finger MYM-type protein 3. Plays a role in the regulation of cell morphology and cytoskeletal organization. May have RNA polymerase II transcription factor activity and

	sequence-specific DNA binding.
ZNF28	Zinc finger protein 28. Transcription factor.
ZNF415	Zinc finger protein 415. Involved in transcriptional regulation. Transcriptional activity differed among the various isoforms. All isoforms except isoform 3 seem to suppresses the transcriptional activities of AP-1 and p53/TP53
ZNF488	Zinc finger protein 488. May be involved in the negative regulation of transcription. Strongly downregulated (~ 4-fold) in testis of frequently infertile cattleyak (PMID:27865410).
ZNF587B	Zinc finger protein 587B. May be involved in transcriptional regulation.
ZNF680	Zinc finger protein 680. Regulates DNA-templated transcription.
ZNF7	Zinc finger protein 7. May be involved in transcriptional regulation. May regulate multicellular organism development. Expressed in human adult testis (PMID:7959769). May regulate activation on non-canonical NF-kB pathway (PMID:24008839).
ZNF773	Zinc finger protein 773. May regulate DNA-templated transcription.
ZSCAN31	Zinc finger and SCAN domain-containing protein 31. May function as a transcription factor. May be involved in the development of multiple embryonic organs.

Table S1.6. RNA transport, processing, and degradation.

Gene	Protein Function
AFF2	AF4/FMR2 family member 2. RNA-binding protein. Might be involved in alternative splicing regulation through an interaction with G-quartet RNA structure.
CPSF3	Cleavage and polyadenylation specificity factor subunit 3. Component of the cleavage and polyadenylation specificity factor (CPSF) complex that play a key role in pre-mRNA 3'-end formation, recognizing the AAUAAA signal sequence and interacting with poly(A) polymerase and other factors to bring about cleavage and poly(A) addition.
DGCR8	Microprocessor complex subunit DGCR8. Component of the microprocessor complex that acts as a RNA- and heme-binding protein that is involved in the initial step of microRNA (miRNA) biogenesis. Involved in the silencing of embryonic stem cell self-renewal (By similarity).
DHX38	Pre-mRNA-splicing factor ATP-dependent RNA helicase PRP16.
DIS3	Exosome complex exonuclease RRP44. Putative catalytic component of the RNA exosome complex which has 3'->5' exoribonuclease activity and participates in a multitude of cellular RNA processing and degradation events. It seems to be involved in degradation of histone mRNA.
DUS3L	tRNA-dihydrouridine(47) synthase [NAD(P)(+)]-like. Catalyzes the synthesis of dihydrouridine, a modified base found in the D-loop of most tRNAs.
ELAVL3	ELAV-like protein 3. Binds to AU-rich sequences (AREs) of target mRNAs, including VEGF mRNA. May also bind poly-A tracts via RRM 3 (By similarity). May be involved in neuronal differentiation and maintenance.
EXOSC10	Exosome component 10. Putative catalytic component of the RNA exosome complex which has 3'->5' exoribonuclease activity and participates in a multitude of cellular RNA processing and degradation events. May be involved in dosage compensation by inactivation of X chromosome.

INTS2	Integrator complex subunit 2. Component of the Integrator (INT) complex, a complex involved in the small nuclear RNAs (snRNA) U1 and U2 transcription and in their 3'-box-dependent processing. Regulates snRNA transcription from RNA polymerase II promoter.
PATL1	Protein PAT1 homolog 1. RNA-binding protein involved in deadenylation-dependent decapping of mRNAs, leading to the degradation of mRNAs. Acts as a scaffold protein that connects deadenylation and decapping machinery.
POLR1B	DNA-directed RNA polymerase I subunit RPA2. DNA-dependent RNA polymerase catalyzes the transcription of DNA into RNA using the four ribonucleoside triphosphates as substrates. Second largest core component of RNA polymerase I which synthesizes ribosomal RNA precursors. Involved in regulation of embryo implantation and positive regulation of gene expression, epigenetic.
PUS3	tRNA pseudouridine(38/39) synthase. Formation of pseudouridine at position 39 in the anticodon stem and loop of transfer RNAs.
PXDNL	Peroxidasin-like protein. Isoform PMR1: Endonuclease selectively degrading some target mRNAs while they are engaged by translating ribosomes, among which albumin and beta-globin mRNAs.
QTRTD1	Queuine tRNA-ribosyltransferase accessory subunit 2. Non-catalytic subunit of the queuine tRNA-ribosyltransferase (TGT).
RAVER1	Ribonucleoprotein PTB-binding 1. Cooperates with PTBP1 to modulate regulated alternative splicing events. Promotes exon skipping. Cooperates with PTBP1 to modulate switching between mutually exclusive exons during maturation of the TPM1 pre-mRNA (By similarity).
RBMX	RNA-binding motif protein, X chromosome. RNA-binding protein that plays several roles in the regulation of pre- and post-transcriptional processes. Regulates mRNA splicing both positively and negatively.
RC3H1	Roquin-1. Post-transcriptional repressor of mRNAs containing a conserved stem loop motif, called constitutive decay element (CDE), which is often located in the 3'-UTR, as in HMGXB3, ICOS, IER3, NFKBID, NFKBIZ, PPP1R10, TNF and in many more mRNAs (By similarity). Metal ion binding. Lymph node development. Spleen development. Regulates immunity and inflammation.
RCL1	RNA 3'-terminal phosphate cyclase-like protein. Does not have cyclase activity. Plays a role in 40S-ribosomal-subunit biogenesis in the early pre-rRNA processing steps at sites A0, A1 and A2 that are required for proper maturation of the 18S RNA (By similarity).
RPRD1A	Regulation of nuclear pre-mRNA domain-containing protein 1A. Interacts with phosphorylated C-terminal heptapeptide repeat domain (CTD) of the largest RNA polymerase II subunit POLR2A, and participates in dephosphorylation of the CTD by RPAP2. May act as a negative regulator of cyclin-D1 (CCND1) and cyclin-E (CCNE1) in the cell cycle.
SCAF4	Splicing factor, arginine/serine-rich 15. May act to physically and functionally link transcription and pre-mRNA processing.
SUPT5H	Transcription elongation factor SPT5. Component of the DRB sensitivity-inducing factor complex (DSIF complex), which regulates mRNA processing and transcription elongation by RNA polymerase II.

TDRD3	Tudor domain-containing protein 3. Scaffolding protein that specifically recognizes and binds dimethylarginine-containing proteins. In nucleus, acts as a coactivator: recognizes and binds asymmetric dimethylation on the core histone tails associated with transcriptional activation (H3R17me2a and H4R3me2a) and recruits proteins at these arginine-methylated loci. In cytoplasm, may play a role in the assembly and/or disassembly of mRNA stress granules and in the regulation of translation of target mRNAs by binding Arg/Gly-rich motifs (GAR) in dimethylarginine-containing proteins. Transcriptional coactivator.
TGS1	Trimethylguanosine synthase. Catalyzes the 2 serial methylation steps for the conversion of the 7-monomethylguanosine (m7G) caps of snRNAs and snoRNAs to a 2,2,7-trimethylguanosine (m(2,2,7)G) cap structure.
TNRC6C	Trinucleotide repeat-containing gene 6C protein. Plays a role in RNA-mediated gene silencing by micro-RNAs (miRNAs). Required for miRNA-dependent translational repression of complementary mRNAs by argonaute family proteins.
TRIT1	tRNA dimethylallyltransferase, mitochondrial. Catalyzes the transfer of a dimethylallyl group onto the adenine at position 37 of both cytosolic and mitochondrial tRNAs, leading to the formation of N6-(dimethylallyl)adenosine (i6A).
TRMT2B	tRNA (uracil(54)-C(5))-methyltransferase homolog. Probable S-adenosyl-L-methionine-dependent methyltransferase that catalyzes the formation of 5-methyl-uridine at position 54 (m5U54) in all tRNA.
URB1	Nucleolar pre-ribosomal-associated protein 1. Binds RNA.
UTP4	U3 small nucleolar RNA-associated protein 4 homolog. Ribosome biogenesis factor. Involved in nucleolar processing of pre-18S ribosomal RNA. Involved in small subunit (SSU) pre-rRNA processing at sites A', A0, 1 and 2b.
XPOT	Exportin-T. Mediates the nuclear export of aminoacylated tRNAs.
XRN2	5'-3' exoribonuclease 2. Possesses 5'->3' exoribonuclease activity (By similarity). May promote the termination of transcription by RNA polymerase II.

Table S1.7. Protein expression, modification, transport, degradation.

Gene	Protein Function
AP2A2	AP-2 complex subunit alpha-2. Component of the adaptor protein complex 2 (AP-2). Adaptor protein complexes function in protein transport via transport vesicles in different membrane traffic pathways.
EEF2K	Eukaryotic elongation factor 2 kinase. Threonine kinase that regulates protein synthesis by controlling the rate of peptide chain elongation. Upon activation by a variety of upstream kinases including AMPK or TRPM7, phosphorylates the elongation factor EEF2 at a single site, renders it unable to bind ribosomes and thus inactive. In turn, the rate of protein synthesis is reduced.
EIF4G3	Eukaryotic translation initiation factor 4 gamma 3. Probable component of the protein complex eIF4F, which is involved in the recognition of the mRNA cap, ATP-dependent unwinding of 5'-terminal secondary structure and recruitment of mRNA to the ribosome.
FAM160A2	FTS and Hook-interacting protein. Component of the FTS/Hook/FHIP complex (FHF complex). The FHF complex may function to promote vesicle trafficking and/or fusion via the homotypic vesicular protein sorting complex (the HOPS complex).
GNL2	Nucleolar GTP-binding protein 2. GTPase that associates with pre-60S ribosomal subunits in the nucleolus and is required for their nuclear export and maturation.

GPR107	Protein GPR107. Involved in Golgi-to-ER retrograde transport. Functions as a host factor required for infection by Pseudomonas aeruginosa exotoxin A and Campylobacter jejuni CDT toxins.
HERC2	E3 ubiquitin-protein ligase HERC2. E3 ubiquitin-protein ligase that regulates ubiquitin-dependent retention of repair proteins on damaged chromosomes.
KHDRBS3	KH domain-containing, RNA-binding, signal transduction-associated protein 3. RNA-binding protein that plays a role in the regulation of alternative splicing and influences mRNA splice site selection and exon inclusion. May play a role as a negative regulator of cell growth. Inhibits cell proliferation.
LMF1	Lipase maturation factor 1. Involved in the maturation of specific proteins in the endoplasmic reticulum. Required for maturation and transport of active lipoprotein lipase (LPL) through the secretory pathway. Each LMF1 molecule chaperones 50 or more molecules of LPL.
LMF2	Lipase maturation factor 2. Involved in the maturation of specific proteins in the endoplasmic reticulum. May be required for maturation and transport of active lipoprotein lipase (LPL) through the secretory pathway (By similarity).
MRPL38	39S ribosomal protein L38, mitochondrial. Regulates mitochondrial translational elongation and termination.
PARP16	Mono [ADP-ribose] polymerase PARP16. Intracellular mono-ADP-ribosyltransferase that may play a role in different processes through the mono-ADP-ribosylation of proteins involved in those processes (PubMed:23103912, PubMed:22701565). May play a role in the unfolded protein response (UPR), by ADP-ribosylating and activating EIF2AK3 and ERN1, two important UPR effectors (PubMed:23103912).
PPIAL4G	Peptidyl-prolyl cis-trans isomerase A-like 4G. PPIases accelerate the folding of proteins. It catalyzes the cis-trans isomerization of proline imidic peptide bonds in oligopeptides (By similarity).
PSMB11	Proteasome subunit beta type-11. The proteasome is a multicatalytic proteinase complex which is characterized by its ability to cleave peptides with Arg, Phe, Tyr, Leu, and Glu adjacent to the leaving group at neutral or slightly basic pH.
RAB3B	Antigen processing and presentation. Positive regulation of dopamine uptake involved in synaptic transmission. Protein transport. Regulation of exocytosis.
RABGAP1L	Rab GTPase-activating protein 1-like. Regulates protein internalization and vesicular fusion.
RANBP6	Ran-binding protein 6. May function in nuclear protein import as nuclear transport receptor.
RCL1	RNA 3'-terminal phosphate cyclase-like protein. Does not have cyclase activity. Plays a role in 40S-ribosomal-subunit biogenesis in the early pre-rRNA processing steps at sites A0, A1 and A2 that are required for proper maturation of the 18S RNA (By similarity).
SEC31A	Protein transport protein Sec31A. Component of the coat protein complex II (COPII) which promotes the formation of transport vesicles from the endoplasmic reticulum (ER) (By similarity).
SENP6	Sentrin-specific protease 6. Protease that deconjugates SUMO1, SUMO2 and SUMO3 from targeted proteins. Regulates kinetochore and spindle assebly.
SPPL2C	Signal peptide peptidase-like 2C. Intramembrane-cleaving aspartic protease (I-CLiP) that may be able to cleave type II membrane signal peptides in the hydrophobic plane of the membrane.
SRP68	Signal recognition particle subunit SRP68. Signal-recognition-particle assembly has a crucial role in targeting secretory proteins to the rough endoplasmic reticulum membrane.

SRP72	Signal recognition particle subunit SRP72. Signal-recognition-particle assembly has a crucial role in targeting secretory proteins to the rough endoplasmic reticulum membrane.
TDRD3	Tudor domain-containing protein 3. Scaffolding protein that specifically recognizes and binds dimethylarginine-containing proteins. In nucleus, acts as a coactivator: recognizes and binds asymmetric dimethylation on the core histone tails associated with transcriptional activation (H3R17me2a and H4R3me2a) and recruits proteins at these arginine-methylated loci. In cytoplasm, may play a role in the assembly and/or disassembly of mRNA stress granules and in the regulation of translation of target mRNAs by binding Arg/Gly-rich motifs (GAR) in dimethylarginine-containing proteins. Transcriptional coactivator.
TGM1	Protein-glutamine gamma-glutamyltransferase K. Catalyzes the cross-linking of proteins and the conjugation of polyamines to proteins. Responsible for cross-linking epidermal proteins during formation of the stratum corneum. Involved in cell proliferation (PubMed:26220141). May positively regulate cell cycle.
TMEM115	Transmembrane protein 115. May play a role in retrograde transport of proteins from the Golgi to the endoplasmic reticulum. May indirectly play a role in protein glycosylation in the Golgi.
TOMM40	Mitochondrial import receptor subunit TOM40 homolog. Channel-forming protein essential for import of protein precursors into mitochondria.
TOR1A	Torsin-1A. Protein with chaperone functions important for the control of protein folding, processing, stability and localization as well as for the reduction of misfolded protein aggregates.
TPP2	Tripeptidyl-peptidase 2. Component of the proteolytic cascade acting downstream of the 26S proteasome in the ubiquitin-proteasome pathway. May be able to complement the 26S proteasome function to some extent under conditions in which the latter is inhibited. Stimulates adipogenesis (By similarity).
TPST1	Protein-tyrosine sulfotransferase 1. Catalyzes the O-sulfation of tyrosine residues within acidic motifs of polypeptides.
UBE4B	Ubiquitin conjugation factor E4 B. Ubiquitin-protein ligase that probably functions as an E3 ligase in conjunction with specific E1 and E2 ligases. May regulate ventricular trabecular myocardium morphogenesis.
UBLCP1	Ubiquitin-like domain-containing CTD phosphatase 1. Dephosphorylates 26S nuclear proteasomes, thereby decreasing their proteolytic activity. The dephosphorylation may prevent assembly of the core and regulatory particles (CP and RP) into mature 26S proteasome.
UBR4	E3 ubiquitin-protein ligase UBR4. E3 ubiquitin-protein ligase which is a component of the N-end rule pathway. Recognizes and binds to proteins bearing specific N-terminal residues that are destabilizing according to the N-end rule, leading to their ubiquitination and subsequent degradation.
UCHL3	Ubiquitin carboxyl-terminal hydrolase isozyme L3. Deubiquitinating enzyme (DUB) that controls levels of cellular ubiquitin through processing of ubiquitin precursors and ubiquitinated proteins.
UFL1	E3 UFM1-protein ligase 1. E3 protein ligase that mediates ufmylation, the covalent attachment of the ubiquitin-like modifier UFM1 to substrate proteins, a post-translational modification on lysine residues of proteins that may play a crucial role in a number of cellular processes.

UGGT1	UDP-glucose:glycoprotein glucosyltransferase 1. Recognizes glycoproteins with minor folding defects. Reglucosylates single N-glycans near the misfolded part of the protein, thus providing quality control for protein folding in the endoplasmic reticulum.
VPS13A	Vacuolar protein sorting-associated protein 13A. May play a role in the control of protein cycling through the trans-Golgi network to early and late endosomes, lysosomes and plasma membrane.
VPS13B	Vacuolar protein sorting-associated protein 13B. May be involved in protein sorting in post Golgi membrane traffic.
XPNPEP1	Xaa-Pro aminopeptidase 1. Contributes to the degradation of bradykinin. Catalyzes the removal of a penultimate prolyl residue from the N-termini of peptides, such as Arg-Pro-Pro.
XPNPEP2	Xaa-Pro aminopeptidase 2. Membrane-bound metalloprotease which catalyzes the removal of a penultimate prolyl residue from the N-termini of peptides, such as Arg-Pro-Pro.

Table S1.8. Membrane proteins, receptors, transporters, and ion channels.

Gene	Protein Function
ABCB1	Multidrug resistance protein 1. Energy-dependent efflux pump responsible for decreased drug accumulation in multidrug-resistant cells.
ABCD1	ATP-binding cassette sub-family D member 1. Probable transporter. The nucleotide-binding fold acts as an ATP-binding subunit with ATPase activity.
ABCG1	ATP-binding cassette sub-family G member 1. Transporter involved in macrophage lipid homeostasis. Is an active component of the macrophage lipid export complex.
ADAM2	Disintegrin and metalloproteinase domain-containing protein 2. Sperm surface membrane protein that may be involved in sperm-egg plasma membrane adhesion and fusion during fertilization.
ATP2B3	Plasma membrane calcium-transporting ATPase 3. This magnesium-dependent enzyme catalyzes the hydrolysis of ATP coupled with the transport of calcium out of the cell.
ATP2C2	Calcium-transporting ATPase type 2C member 2. This magnesium-dependent enzyme catalyzes the hydrolysis of ATP coupled with the transport of calcium. Metal ion binding.
ATP8A2	Phospholipid-transporting ATPase IB. Catalytic component of a P4-ATPase flippase complex which catalyzes the hydrolysis of ATP coupled to the transport of aminophospholipids from the outer to the inner leaflet of various membranes and ensures the maintenance of asymmetric distribution of phospholipids.
CACNA1D	Voltage-dependent L-type calcium channel subunit alpha-1D. Voltage-sensitive calcium channels (VSCC) mediate the entry of calcium ions into excitable cells and are also involved in a variety of calcium-dependent processes, including muscle contraction, hormone or neurotransmitter release, gene expression, cell motility, cell division and cell death.
CACNB3	Voltage-dependent L-type calcium channel subunit beta-3.
CMTM8	CKLF-like MARVEL transmembrane domain-containing protein 8. Structural component of myelin sheath. May have cytokine activity.
DCC	Netrin receptor DCC. Receptor for netrin required for axon guidance.
FRAS1	Extracellular matrix protein FRAS1. Metal ion binding. Participates in embryonic limb, metanephros, and epithelium morphogeneses.
GLP2R	Glucagon-like peptide 2 receptor. This is a receptor for glucagon-like peptide 2. The activity of this receptor is mediated by G proteins which activate adenylyl cyclase.

GLRA1	Glycine receptor subunit alpha-1. Glycine receptors are ligand-gated chloride channels (PubMed:23994010, PubMed:25730860).
GNL2	Nucleolar GTP-binding protein 2. GTPase that associates with pre-60S ribosomal subunits in the nucleolus and is required for their nuclear export and maturation.
GPC1	Glypican-1. Cell surface proteoglycan that bears heparan sulfate. Binds, via the heparan sulfate side chains, alpha-4 (V) collagen and participates in Schwann cell myelination (By similarity). May negatively regulate FGFR signaling.
GPR89A	Golgi pH regulator A. Voltage dependent anion channel required for acidification and functions of the Golgi apparatus that may function in counter-ion conductance.
HPSE2	Inactive heparanase-2. Binds heparin and heparan sulfate with high affinity, but lacks heparanase activity. Inhibits HPSE, possibly by competing for its substrates (in vitro).
ITPR2	Inositol 1,4,5-trisphosphate receptor type 2. Receptor for inositol 1,4,5-trisphosphate, a second messenger that mediates the release of intracellular calcium. This release is regulated by cAMP both dependently and independently of PKA. May regulate cellular response to hypoxia.
KCNA10	Potassium voltage-gated channel subfamily A member 10. Mediates voltage-dependent potassium ion permeability of excitable membranes.
KCNB2	Potassium voltage-gated channel subfamily B member 2. Voltage-gated potassium channel that mediates transmembrane potassium transport in excitable membranes, primarily in the brain and smooth muscle cells. Regulates smooth muscle cell contraction.
KPNB1	Importin subunit beta-1. Functions in nuclear protein import, either in association with an adapter protein, like an importin-alpha subunit, which binds to nuclear localization signals (NLS) in cargo substrates, or by acting as autonomous nuclear transport receptor.
LRP1B	Low-density lipoprotein receptor-related protein 1B. Potential cell surface proteins that bind and internalize ligands in the process of receptor-mediated endocytosis. The gene is preferentially inactivated in one histological type of lung cancer (non-small cell lung cancer (NSCLC)).
LTF	Lactotransferrin. Transferrins are iron binding transport proteins which can bind two Fe^{3+} ions in association with the binding of an anion, usually bicarbonate. Regulates innate and adaptive immunity as well as inflammation.
MUC2	Mucin-2. Coats the epithelia of the intestines, airways, and other mucus membrane-containing organs.
MUC5B	Mucin-5B. Gel-forming mucin that is thought to contribute to the lubricating and viscoelastic properties of whole saliva and cervical mucus.
NCAN	Neurocan core protein. May modulate neuronal adhesion and neurite growth during development by binding to neural cell adhesion molecules (NG-CAM and N-CAM). Chondroitin sulfate proteoglycan; binds to hyaluronic acid. May regulate skeletal system development.
NIPAL3	Magnesium ion transporter
NPHS2	Podocin. Plays a role in the regulation of glomerular permeability, acting probably as a linker between the plasma membrane and the cytoskeleton.
NUP153	Nuclear pore complex protein Nup153. Component of the nuclear pore complex (NPC), a complex required for the trafficking across the nuclear envelope.
PGAP2	Post-GPI attachment to proteins factor 2. Involved in the lipid remodeling steps of GPI-anchor maturation. Required for stable expression of GPI-anchored proteins at the cell surface (By similarity).
PMP2	Myelin P2 protein. May play a role in lipid transport protein in Schwann cells. May bind

	cholesterol.
PRELID3B	PRELI domain containing protein 3B. Has phosphatidic acid transporter activity.
PTGIR	Prostacyclin receptor. Receptor for prostacyclin (prostaglandin I2 or PGI2). The activity of this receptor is mediated by G(s) proteins which activate adenylate cyclase.
RBP3	Retinol-binding protein 3. IRBP shuttles 11-cis and all trans retinoids between the retinol isomerase in the pigment epithelium and the visual pigments in the photoreceptor cells of the retina.
RYR2	Ryanodine receptor 2. Calcium channel that mediates the release of Ca2+ from the sarcoplasmic reticulum into the cytoplasm and thereby plays a key role in triggering cardiac muscle contraction. Involved in BMP signaling pathway.
SCARF2	Scavenger receptor class F member 2. Probable adhesion protein, which mediates homophilic and heterophilic interactions. In contrast to SCARF1, it poorly mediates the binding and degradation of acetylated low density lipoprotein (Ac-LDL).
SDC3	Syndecan-3. Cell surface proteoglycan that may bear heparan sulfate (By similarity). May have role in organization of cell shape.
SLC12A5	Solute carrier family 12 member 5. Mediates electroneutral potassium-chloride cotransport in mature neurons and is required for neuronal Cl- homeostasis.
SLC13A2	SLC13A2 protein. Has transporter activity.
SLC22A18	Solute carrier family 22 member 18. May act as a transporter of organic cations based on a proton efflux antiport mechanism. May play a role in the transport of chloroquine and quinidine-related compounds in kidney.
SLC23A2	Solute carrier family 23 member 2. Sodium/ascorbate cotransporter. Mediates electrogenic uptake of vitamin C, with a stoichiometry of 2 Na+ for each ascorbate.
SLC26A2	Sulfate transporter. May play a role in endochondral bone formation.
SLC5A10	Sodium/glucose cotransporter 5. High capacity transporter for mannose and fructose and, to a lesser extent, glucose, AMG, and galactose.
SLC5A4	Sodium glucose transmembrane transporter.
SLC6A5	Sodium- and chloride-dependent glycine transporter 2. Terminates the action of glycine by its high affinity sodium-dependent reuptake into presynaptic terminals.
TAS2R19	Taste receptor type 2 member 19. Receptor that may play a role in the perception of bitterness and is gustducin-linked.
TFRC	Transferrin receptor protein 1. Cellular uptake of iron occurs via receptor-mediated endocytosis of ligand-occupied transferrin receptor into specialized endosomes. Endosomal acidification leads to iron release. The apotransferrin-receptor complex is then recycled to the cell surface with a return to neutral pH and the concomitant loss of affinity of apotransferrin for its receptor.
TMEM54	Transmembrane protein 54.
TMEM8A	Transmembrane protein 8A. May be a cell surface adhesion molecule. May be involved in the maintenance of the resting T-cell state.
TRIM45	Tripartite motif-containing protein 45. Zinc ion binding. Bone development.
XPOT	Exportin-T. Mediates the nuclear export of aminoacylated tRNAs.
ZSWIM5	Zinc finger SWIM domain-containing protein 5. Zinc ion binding.

Table S1.9. Immunity and inflammation.

Gene	Protein Function
ABCF3	ATP-binding cassette sub-family F member 3. Displays an antiviral effect against flaviviruses such as west Nile virus (WNV) in the presence of OAS1B.
ACVR1B	Activin receptor type-1B. Transmembrane serine/threonine kinase activin type-1 receptor forming an activin receptor complex with activin receptor type-2 (ACVR2A or ACVR2B). Regulates neuronal differentiation and neuronal survival, hair follicle development and cycling, FSH production by the pituitary gland, wound healing, extracellular matrix production, immunosuppression and carcinogenesis. May regulate development of primary female sexual characteristics and in utero embryonic development.
ANXA1	Annexin A1. Plays important roles in the innate immune response as effector of glucocorticoid-mediated responses and regulator of the inflammatory process. Promotes resolution of inflammation and wound healing (PubMed:25664854).
CCL13	C-C motif chemokine 13. Chemotactic factor that attracts monocytes, lymphocytes, basophils and eosinophils, but not neutrophils. May regulate animal organ regeneration and intracellular signaling.
CCR6	C-C chemokine receptor type 6. Receptor for the C-C type chemokine CCL20 (PubMed:9169459). Regulates cell chemotaxis and humoral immune response.
CD74	HLA class II histocompatibility antigen gamma chain. Plays a critical role in MHC class II antigen processing by stabilizing peptide-free class II alpha/beta heterodimers in a complex soon after their synthesis and directing transport of the complex from the endoplasmic reticulum to the endosomal/lysosomal system where the antigen processing and binding of antigenic peptides to MHC class II takes place. Serves as cell surface receptor for the cytokine MIF.
CMTM8	CKLF-like MARVEL transmembrane domain-containing protein 8. Structural component of myelin sheath. May have cytokine activity.
CYFIP2	Cytoplasmic FMR1-interacting protein 2. Involved in T-cell adhesion and p53/TP53-dependent induction of apoptosis. As component of the WAVE1 complex, required for BDNF-NTRK2 endocytic trafficking and signaling from early endosomes (By similarity).
DEFA4	Neutrophil defensin 4. Has antimicrobial activity against Gram-negative bacteria, and to a lesser extent also against Gram-positive bacteria and fungi.
ERAP2	Endoplasmic reticulum aminopeptidase 2. Aminopeptidase that plays a central role in peptide trimming, a step required for the generation of most HLA class I-binding peptides.
GGT5	Gamma-glutamyltransferase 5. Cleaves the gamma-glutamyl peptide bond of glutathione conjugates, but maybe not glutathione itself. Converts leukotriene C4 (LTC4) to leukotriene D4 (LTD4).
GNAQ	Guanine nucleotide-binding protein G(q) subunit alpha. Guanine nucleotide-binding proteins (G proteins) are involved as modulators or transducers in various transmembrane signaling systems. Regulates B-cell selection and survival and is required to prevent B-cell-dependent autoimmunity. Regulates chemotaxis of BM-derived neutrophils and dendritic cells (in vitro) (By similarity).
GPR107	Protein GPR107. Involved in Golgi-to-ER retrograde transport. Functions as a host factor required for infection by Pseudomonas aeruginosa exotoxin A and Campylobacter jejuni CDT toxins.
IL17B	Interleukin-17B. Stimulates the release of tumor necrosis factor alpha and IL-1-beta from the monocytic cell line THP-1. Positively regulates cytokine production involved in the

	inflammatory response.
ITGAL	Integrin alpha-L. Integrin alpha-L/beta-2 is a receptor for ICAM1, ICAM2, ICAM3 and ICAM4. Integrin alpha-L/beta-2 is also a receptor for F11R (PubMed:11812992, PubMed:15528364). Involved in a variety of immune phenomena including leukocyte-endothelial cell interaction, cytotoxic T-cell mediated killing, and antibody dependent killing by granulocytes and monocytes.
ITGAX	Integrin alpha-X. Integrin alpha-X/beta-2 is a receptor for fibrinogen. It recognizes the sequence G-P-R in fibrinogen. It mediates cell-cell interaction during inflammatory responses. It is especially important in monocyte adhesion and chemotaxis. May regulate animal organ morphogenesis.
LTF	Lactotransferrin. Transferrins are iron binding transport proteins which can bind two Fe^{3+} ions in association with the binding of an anion, usually bicarbonate. Regulates innate and adaptive immunity as well as inflammation.
NFATC2IP	NFATC2-interacting protein. In T-helper 2 (Th2) cells, regulates the magnitude of NFAT-driven transcription of a specific subset of cytokine genes, including IL3, IL4, IL5 and IL13, but not IL2. Recruits PRMT1 to the IL4 promoter; this leads to enhancement of histone H4 'Arg-3'-methylation and facilitates subsequent histone acetylation at the IL4 locus, thus promotes robust cytokine expression (By similarity).
NFKB1	Nuclear factor NF-kappa-B p105 subunit. NF-kappa-B is a pleiotropic transcription factor present in almost all cell types and is the endpoint of a series of signal transduction events that are initiated by a vast array of stimuli related to many biological processes such as inflammation, immunity, differentiation, cell growth, tumorigenesis and apoptosis. Also involved in regulation of transcription from RNA polymerase II promoter.
NR3C1	Glucocorticoid receptor. Receptor for glucocorticoids (GC) (PubMed:27120390). Has a dual mode of action: as a transcription factor that binds to glucocorticoid response elements (GRE), both for nuclear and mitochondrial DNA, and as a modulator of other transcription factors. Affects inflammatory responses, cellular proliferation and differentiation in target tissues.
RAB3B	Antigen processing and presentation. Positive regulation of dopamine uptake involved in synaptic transmission. Protein transport. Regulation of exocytosis.
RC3H1	Roquin-1. Post-transcriptional repressor of mRNAs containing a conserved stem loop motif, called constitutive decay element (CDE), which is often located in the 3'-UTR, as in HMGXB3, ICOS, IER3, NFKBID, NFKBIZ, PPP1R10, TNF and in many more mRNAs (By similarity). Metal ion binding. Lymph node development. Spleen development. Regulates immunity and inflammation.
SCRN1	Secernin-1. Regulates exocytosis in mast cells. Increases both the extent of secretion and the sensitivity of mast cells to stimulation with calcium (By similarity).
SEC14L1	SEC14-like protein 1. May play a role in innate immunity by inhibiting the antiviral RIG-I signaling pathway. In this pathway, functions as a negative regulator of DDX58/RIG-I, the cytoplasmic sensor of viral nucleic acids. May also regulate the SLC18A3 and SLC5A7 cholinergic transporters.
STK10	Serine/threonine-protein kinase 10. Serine/threonine-protein kinase involved in regulation of lymphocyte migration.
TMEM8A	Transmembrane protein 8A. May be a cell surface adhesion molecule. May be involved in the maintenance of the resting T-cell state.

TNFAIP8L2	Tumor necrosis factor alpha-induced protein 8-like protein 2. Acts as a negative regulator of innate and adaptive immunity by maintaining immune homeostasis. Negative regulator of Toll-like receptor and T-cell receptor function. Prevents hyperresponsiveness of the immune system and maintains immune homeostasis. Inhibits JUN/AP1 and NF-kappa-B activation. Promotes Fas-induced apoptosis.
ZNF7	Zinc finger protein 7. May be involved in transcriptional regulation. May regulate multicellular organism development. Expressed in human adult testis (PMID:7959769). May regulate activation on non-canonical NF-kB pathway (PMID:24008839).

Table S1.10. Neuron differentiation, migration, and neurotransmission.

Gene	Protein Function
ACVR1B	Activin receptor type-1B. Transmembrane serine/threonine kinase activin type-1 receptor forming an activin receptor complex with activin receptor type-2 (ACVR2A or ACVR2B). Regulates neuronal differentiation and neuronal survival, hair follicle development and cycling, FSH production by the pituitary gland, wound healing, extracellular matrix production, immunosuppression and carcinogenesis. May regulate development of primary female sexual characteristics and in utero embryonic development.
AFG3L2	AFG3-like protein 2. ATP-dependent protease which is essential for axonal and neuron development. May regulate muscle fiber development.
CACNA1D	Voltage-dependent L-type calcium channel subunit alpha-1D. Voltage-sensitive calcium channels (VSCC) mediate the entry of calcium ions into excitable cells and are also involved in a variety of calcium-dependent processes, including muscle contraction, hormone or neurotransmitter release, gene expression, cell motility, cell division and cell death.
CADM2	Cell adhesion molecule 2. Adhesion molecule that engages in homo- and heterophilic interactions with the other nectin-like family members, leading to cell aggregation. Important for synapse organization.
CAMSAP1	Calmodulin-regulated spectrin-associated protein 1. Probable microtubule-binding protein that plays a role in the regulation of cell morphology and cytoskeletal organization. Through interaction with spectrin may regulate neurite outgrowth.
CASZ1	Zinc finger protein castor homolog 1. Transcriptional activator (PubMed:23639441, PubMed:27693370). Involved in vascular assembly and morphogenesis through direct transcriptional regulation of EGFL7 (PubMed:23639441). May also regulate neuron differentiation.
CNTN5	Contactin-5. Contactins mediate cell surface interactions during nervous system development.
CSMD3	CUB and sushi domain-containing protein 3. Involved in dendrite development.
CTTNBP2	Cortactin-binding protein 2. Regulates the dendritic spine distribution of CTTN/cortactin in hippocampal neurons, thus controls dendritic spinogenesis and dendritic spine maintenance.
DCC	Netrin receptor DCC. Receptor for netrin required for axon guidance.
DYSF	Dysferlin. Key calcium ion sensor involved in the Ca2+-triggered synaptic vesicle-plasma membrane fusion. Plays a role in the sarcolemma repair mechanism of both skeletal muscle and cardiomyocytes that permits rapid resealing of membranes disrupted by mechanical stress (By similarity).
ELAVL3	ELAV-like protein 3. Binds to AU-rich sequences (AREs) of target mRNAs, including VEGF

	mRNA. May also bind poly-A tracts via RRM 3 (By similarity). May be involved in neuronal differentiation and maintenance.
GPR12	G-protein coupled receptor 12. Promotes neurite outgrowth and blocks myelin inhibition in neurons (By similarity). Receptor with constitutive G(s) signaling activity that stimulates cyclic AMP production.
GPRIN2	G protein-regulated inducer of neurite outgrowth 2. May be involved in neurite outgrowth.
IGSF10	Immunoglobulin superfamily member 10. Involved in the control of early migration of neurons expressing gonadotropin-releasing hormone (GNRH neurons) (By similarity). May be involved in the maintenance of osteochondroprogenitor cells pool (By similarity).
KIF13B	Kinesin-like protein KIF13B. Involved in reorganization of the cortical cytoskeleton.
MYO5A	Unconventional myosin-Va. Processive actin-based motor that can move in large steps approximating the 36-nm pseudo-repeat of the actin filament. Involved in melanosome transport. Also mediates the transport of vesicles to the plasma membrane. May also be required for some polarization process involved in dendrite formation.
NCAN	Neurocan core protein. May modulate neuronal adhesion and neurite growth during development by binding to neural cell adhesion molecules (NG-CAM and N-CAM). Chondroitin sulfate proteoglycan; binds to hyaluronic acid. May regulate skeletal system development.
NEFH	Neurofilament heavy polypeptide. Neurofilaments usually contain three intermediate filament proteins: L, M, and H which are involved in the maintenance of neuronal caliber.
NELL2	Protein kinase C-binding protein NELL2. Required for neuron survival through the modulation of MAPK pathways (By similarity). Involved in the regulation of hypothalamic GNRH secretion and the control of puberty (By similarity).
NHSL1	NHS-like protein 1. May regulate motor neuron migration.
OPHN1	Oligophrenin-1. Stimulates GTP hydrolysis of members of the Rho family. Its action on RHOA activity and signaling is implicated in growth and stabilization of dendritic spines, and therefore in synaptic function.
PCDHGC5	Protocadherin gamma-C5. Potential calcium-dependent cell-adhesion protein. May be involved in the establishment and maintenance of specific neuronal connections in the brain.
SEC14L1	SEC14-like protein 1. May play a role in innate immunity by inhibiting the antiviral RIG-I signaling pathway. In this pathway, functions as a negative regulator of DDX58/RIG-I, the cytoplasmic sensor of viral nucleic acids. May also regulate the SLC18A3 and SLC5A7 cholinergic transporters.
SHANK3	SH3 and multiple ankyrin repeat domains protein 3. Major scaffold postsynaptic density protein which interacts with multiple proteins and complexes to orchestrate the dendritic spine and synapse formation, maturation and maintenance.
SLC12A5	Solute carrier family 12 member 5. Mediates electroneutral potassium-chloride cotransport in mature neurons and is required for neuronal Cl- homeostasis.
SLC6A5	Sodium- and chloride-dependent glycine transporter 2. Terminates the action of glycine by its high affinity sodium-dependent reuptake into presynaptic terminals.
TENM4	Teneurin-4. Involved in neural development, regulating the establishment of proper connectivity within the nervous system. Plays a role in the establishment of the anterior-posterior axis during gastrulation.

Table S1.11. Carcinogenesis/Tumor suppression.

Gene	Protein Function
ACVR1B	Activin receptor type-1B. Transmembrane serine/threonine kinase activin type-1 receptor forming an activin receptor complex with activin receptor type-2 (ACVR2A or ACVR2B). Regulates neuronal differentiation and neuronal survival, hair follicle development and cycling, FSH production by the pituitary gland, wound healing, extracellular matrix production, immunosuppression and carcinogenesis. May regulate development of primary female sexual characteristics and in utero embryonic development.
APC	Adenomatous polyposis coli protein. Tumor suppressor. Promotes rapid degradation of CTNNB1 and participates in Wnt signaling as a negative regulator.
CAMTA1	Calmodulin-binding transcription activator 1. Transcriptional activator. May act as a tumor suppressor.
CFAP58	Cilia- and flagella-associated protein 58. Formerly known as CCDC147. Fuses with FGFR2 n intra-hepatic cholangiocarcinoma (link) One of the genes associated with atypical femoral fractures (PMID: 26160281).
CRIPAK	Cysteine-rich PAK1 inhibitor. Negative regulator of PAK1. It has been suggested that the loss of CRIPAK in breast tumors might contribute to hormonal independence.
CSMD1	CUB and sushi domain-containing protein 1. Potential suppressor of squamous cell carcinomas.
LRP1B	Low-density lipoprotein receptor-related protein 1B. Potential cell surface proteins that bind and internalize ligands in the process of receptor-mediated endocytosis. The gene is preferentially inactivated in one histological type of lung cancer (non-small cell lung cancer (NSCLC)).
NBEAL1	Neurobeachin-like protein 1. Highly expressed in brain, kidney, prostate and testis. Weakly expressed in ovary, small intestine, colon and peripheral blood leukocytes. May be correlative to several tumors, such as ovary serous adenocarcinoma and metastasis mammary gland carcinoma breast.
NFKB1	Nuclear factor NF-kappa-B p105 subunit. NF-kappa-B is a pleiotropic transcription factor present in almost all cell types and is the endpoint of a series of signal transduction events that are initiated by a vast array of stimuli related to many biological processes such as inflammation, immunity, differentiation, cell growth, tumorigenesis and apoptosis. Also involved in regulation of transcription from RNA polymerase II promoter.
PKM	Pyruvate kinase PKM. Glycolytic enzyme that catalyzes the transfer of a phosphoryl group from phosphoenolpyruvate (PEP) to ADP, generating ATP. Stimulates POU5F1-mediated transcriptional activation. Plays a general role in caspase independent cell death of tumor cells. May regulate animal organ regeneration.
QSOX1	Sulfhydryl oxidase 1. Catalyzes the oxidation of sulfhydryl groups in peptide and protein thiols to disulfides with the reduction of oxygen to hydrogen peroxide. May contribute to disulfide bond formation in a variety of secreted proteins. In fibroblasts, it may have tumor-suppressing capabilities being involved in growth regulation.
TCHP	Trichoplein keratin filament-binding protein. Tumor suppressor which has the ability to inhibit cell growth and be pro-apoptotic during cell stress. Inhibits cell growth in bladder and prostate cancer cells by a down-regulation of HSPB1 by inhibiting its phosphorylation.

USP6	Ubiquitin carboxyl-terminal hydrolase 6. Deubiquitinase with an ATP-independent isopeptidase activity, cleaving at the C-terminus of the ubiquitin moiety. Is able to initiate tumorigenesis by inducing the production of matrix metalloproteinases following NF-kappa-B activation.
WWC1	Protein KIBRA. Probable regulator of the Hippo/SWH (Sav/Wts/Hpo) signaling pathway, a signaling pathway that plays a pivotal role in tumor suppression by restricting proliferation and promoting apoptosis. May negatively regulate organ growth.

Table S1.12. Environmental stress.

Gene	Protein Function
CYP2D6	Cytochrome P450 2D6. Drugs and environmental chemicals metabolism.
GGT6	Gamma-glutamyltransferase 6. Cleaves glutathione conjugates.
HSP90AB1	Heat shock protein HSP 90-beta. Response to environmental change.
OSGIN1	Oxidative stress-induced growth inhibitor 1.

Table S1.13. Miscellaneous genes.

Gene	Protein Function
ABHD1	Enzyme that has carboxylic ester hydrolase and lipase activities.
ACSL3	Long-chain-fatty-acid--CoA ligase 3. Acyl-CoA synthetases (ACSL) activates long-chain fatty acids for both synthesis of cellular lipids, and degradation via beta-oxidation. ACSL3 mediates hepatic lipogenesis (By similarity).
ACSL5	Long-chain-fatty-acid--CoA ligase 5. Acyl-CoA synthetases (ACSL) activate long-chain fatty acids for both synthesis of cellular lipids, and degradation via beta-oxidation. ACSL5 may activate fatty acids from exogenous sources for the synthesis of triacylglycerol destined for intracellular storage (By similarity).
ACSM1	Acyl-coenzyme A synthetase ACSM1, mitochondrial. Has medium-chain fatty acid:CoA ligase activity with broad substrate specificity (in vitro). Acts on acids from C4 to C(11) and on the corresponding 3-hydroxy- and 2,3- or 3,4-unsaturated acids (in vitro).
ADAMTS7	A disintegrin and metalloproteinase with thrombospondin motifs 7. Metalloprotease that may play a role in the degradation of COMP.
ALAS1	5-aminolevulinate synthase, nonspecific, mitochondrial. This protein is involved in step 1 of the subpathway that synthesizes 5-aminolevulinate from glycine.
CAD	This protein is a "fusion" protein encoding four enzymatic activities of the pyrimidine pathway (GATase, CPSase, ATCase and DHOase).
CMYA5	Cardiomyopathy-associated protein 5. May serve as an anchoring protein that mediates the subcellular compartmentation of protein kinase A (PKA) via binding to PRKAR2A (By similarity). May function as a repressor of calcineurin-mediated transcriptional activity. May attenuate calcineurin ability to induce slow-fiber gene program in muscle and may negatively modulate skeletal muscle regeneration (By similarity).
CPS1	Carbamoyl-phosphate synthase [ammonia], mitochondrial. Involved in the urea cycle of ureotelic animals where the enzyme plays an important role in removing excess ammonia from the cell.

CYP1A1	Cytochrome P450 1A1. Cytochromes P450 are a group of heme-thiolate monooxygenases. In liver microsomes, this enzyme is involved in an NADPH-dependent electron transport pathway. It oxidizes a variety of structurally unrelated compounds, including steroids, fatty acids, and xenobiotics.
CYP2B6	Cytochrome P450 2B6. Cytochromes P450 are a group of heme-thiolate monooxygenases. In liver microsomes, this enzyme is involved in an NADPH-dependent electron transport pathway. It oxidizes a variety of structurally unrelated compounds, including steroids, fatty acids, and xenobiotics.
CYP4X1	Cytochrome P450 4X1. Has aromatase activity. Binds heme and iron.
DPYD	Dihydropyrimidine dehydrogenase [NADP(+)]. This protein is involved in the pathway beta-alanine biosynthesis, which is part of Amino-acid biosynthesis. Involved in pyrimidine base degradation. Catalyzes the reduction of uracil and thymine.
ETNK2	Ethanolamine kinase 2. Highly specific for ethanolamine phosphorylation. Does not have choline kinase activity (By similarity). May regulate multicellular organism growth and placenta development.
GAST	Gastrin. Gastrin stimulates the stomach mucosa to produce and secrete hydrochloric acid and the pancreas to secrete its digestive enzymes.
GCNT3	Beta-1,3-galactosyl-O-glycosyl-glycoprotein beta-1,6-N-acetylglucosaminyltransferase 3. Glycosyltransferase that can synthesize all known mucin beta 6 N-acetylglucosaminides.
GGT6	Gamma-glutamyltransferase 6. Cleaves glutathione conjugates.
HSD17B4	Peroxisomal multifunctional enzyme type 2. Bifunctional enzyme acting on the peroxisomal beta-oxidation pathway for fatty acids.
KRT33B	Keratin, type I cuticular Ha3-II. There are two types of hair/microfibrillar keratin, I (acidic) and II (neutral to basic).
KRT37	Keratin, type I cuticular Ha7.
KRTAP26-1	Keratin-associated protein 26-1. In the hair cortex, hair keratin intermediate filaments are embedded in an interfilamentous matrix, consisting of hair keratin-associated proteins (KRTAP), which are essential for the formation of a rigid and resistant hair shaft through their extensive disulfide bond cross-linking with abundant cysteine residues of hair keratins.
KRTAP4-9	Keratin-associated protein 4-9. In the hair cortex, hair keratin intermediate filaments are embedded in an interfilamentous matrix, consisting of hair keratin-associated proteins (KRTAP), which are essential for the formation of a rigid and resistant hair shaft through their extensive disulfide bond cross-linking with abundant cysteine residues of hair keratins.
LACE1	Lactation elevated 1 also known as AFG1L (AFG1-like ATPase). Putative mitochondrial ATPase. Plays a role in mitochondrial morphology and mitochondrial protein metabolism.
LGALS4	Galectin-4. Galectin that binds lactose and a related range of sugars. May be involved in the assembly of adherens junctions.
LMF2	Lipase maturation factor 2. Involved in the maturation of specific proteins in the endoplasmic reticulum. May be required for maturation and transport of active lipoprotein lipase (LPL) through the secretory pathway (By similarity).
MOCS1	Molybdenum cofactor biosynthesis protein 1. This protein is involved in the pathway molybdopterin biosynthesis, which is part of cofactor biosynthesis.
NOM1	Nucleolar MIF4G domain-containing protein 1. Plays a role in targeting PPP1CA to the nucleolus. May regulate hair follicle maturation.

NOX3	NADPH oxidase 3. NADPH oxidase which constitutively produces superoxide upon formation of a complex with CYBA/p22phox.
NUDT17	Nucleoside diphosphate-linked moiety X motif 17. Probably mediates the hydrolysis of some nucleoside diphosphate derivatives.
PKM	Pyruvate kinase PKM. Glycolytic enzyme that catalyzes the transfer of a phosphoryl group from phosphoenolpyruvate (PEP) to ADP, generating ATP. Stimulates POU5F1-mediated transcriptional activation. Plays a general role in caspase independent cell death of tumor cells. May regulate animal organ regeneration.
PKP2	Plakophilin-2. May play a role in junctional plaques. May be involved in the maintenance of animal organ identity.
PLD3	Phospholipase D3. May be involved in APP processing.
PPIAL4G	Peptidyl-prolyl cis-trans isomerase A-like 4G. PPIases accelerate the folding of proteins. It catalyzes the cis-trans isomerization of proline imidic peptide bonds in oligopeptides (By similarity).
PPIL4	Peptidyl-prolyl cis-trans isomerase-like 4. PPIases accelerate the folding of proteins. It catalyzes the cis-trans isomerization of proline imidic peptide bonds in oligopeptides (By similarity).
PPT2	Lysosomal thioesterase PPT2. Removes thioester-linked fatty acyl groups from various substrates including S-palmitoyl-CoA. Participates in fatty-acyl-CoA biosynthetic process.
PRELID3B	PRELI domain containing protein 3B. Transports phosphatidic acid.
SRPRA	Signal recognition particle receptor subunit alpha. Component of the SRP (signal recognition particle) receptor. Ensures, in conjunction with the signal recognition particle, the correct targeting of the nascent secretory proteins to the endoplasmic reticulum membrane system.
ST6GALNAC4	Alpha-N-acetyl-neuraminyl-2,3-beta-galactosyl-1,3-N-acetyl-galactosaminide alpha-2,6-sialyltransferase. Involved in the biosynthesis of ganglioside GD1A from GM1B.
SULF2	Extracellular sulfatase Sulf-2. Exhibits arylsulfatase activity and highly specific endoglucosamine-6-sulfatase activity. It can remove sulfate from the C-6 position of glucosamine within specific subregions of intact heparin.
SYNJ2	Synaptojanin-2. Inhibits effect of Rac1 on endocytosis.
TGM3	Protein-glutamine gamma-glutamyltransferase E. Catalyzes the calcium-dependent formation of isopeptide cross-links between glutamine and lysine residues in various proteins, as well as the conjugation of polyamines to proteins. Involved in the formation of the cornified envelope (CE), a specialized component consisting of covalent cross-links of proteins beneath the plasma membrane of terminally differentiated keratinocytes. In hair follicles, involved in cross-linking structural proteins to hardening the inner root sheath.
TTLL4	Tubulin polyglutamylase TTLL4. Glutamylase which preferentially modifies beta-tubulin and non-tubulin proteins, such as NAP1L1, NAP1L4 and CGAS/MB21D1.
TTN	Titin. Key component in the assembly and functioning of vertebrate striated muscles. By providing connections at the level of individual microfilaments, it contributes to the fine balance of forces between the two halves of the sarcomere.
XDH	Xanthine dehydrogenase/oxidase. Key enzyme in purine degradation. Catalyzes the oxidation of hypoxanthine to xanthine. Catalyzes the oxidation of xanthine to uric acid. Contributes to the generation of reactive oxygen species.
ZP3	Zona pellucida sperm-binding protein 3. ZP3 is essential for sperm binding and zona matrix formation.

Table S1.14. Uncharacterized or poorly characterized genes.

Gene	Protein Function
2-Mar	
ADGRV1	Adhesion G protein-coupled receptor V1.
ALLC	Probable allantoicase. The function of this enzyme is unclear as allantoicase activity is not known to exist in mammals.
C12orf10	UPF0160 protein MYG1, mitochondrial.
C12orf29	Uncharacterized protein C12orf29.
C1orf100	Uncharacterized protein C1orf100 homolog
C5orf46	Uncharacterized protein C5orf46.
CAMKV	CaM kinase-like vesicle-associated protein. Does not appear to have detectable kinase activity.
CCDC178	Coiled-coil domain-containing protein 178.
CCDC189	Coiled-coil domain-containing protein 189.
CDCP2	CUB domain-containing protein 2.
CPA3	Mast cell carboxypeptidase A. Releases C-terminal amino acid, but little or no action with -Asp, -Glu, -Arg, -Lys or -Pro.
DDX49	Probable ATP-dependent RNA helicase DDX49.
DDX55	ATP-dependent RNA helicase DDX55. Probable ATP-binding RNA helicase.
DMXL1	DmX-like protein 1. May regulate vacuolar acidification.
FAM177A1	Protein FAM177A1.
FAM222A	Protein FAM222A.
FAM227A	Protein FAM227A.
HARS2	Probable histidine--tRNA ligase, mitochondrial. Enzymatic activity: ATP + L-histidine + tRNA(His) = AMP + diphosphate + L-histidyl-tRNA(His).
ISOC2	Isochorismatase domain-containing protein 2.
KCTD18	BTB/POZ domain-containing protein KCTD18.
KDELC1	KDEL motif-containing protein 1. Has glycosyltransferase activity.
KNOP1	Lysine-rich nucleolar protein 1.
LRRC31	Leucine-rich repeat-containing protein 31.
LRRN2	Leucine rich repeat protein 2, neuronal, isoform CRA_b.
METTL15	Probable methyltransferase-like protein 15. Probable S-adenosyl-L-methionine-dependent methyltransferase.
MORN3	MORN repeat-containing protein 3.
NDUFB7	NADH dehydrogenase [ubiquinone] 1 beta subcomplex subunit 7. Accessory subunit of the mitochondrial membrane respiratory chain NADH dehydrogenase (Complex I), that is believed not to be involved in catalysis.
OR10G8	Olfactory receptor family 10 subfamily G member 8,
OR4C46	Olfactory receptor 4C46. Regulates G-protein coupled receptor activity. Detects chemical stimulus involved in sensory perception.
OR4K1	Olfactory receptor family 4 subfamily K member 1
OR4X1	Olfactory receptor family 4 subfamily X member 1.

OR51A4	Olfactory receptor 51A4.
OR51V1	Olfactory receptor 51V1.
OR52E4	Olfactory receptor family 52 subfamily E member 4.
OR52R1	Olfactory receptor family 52 subfamily R member 1.
OR5C1	Olfactory receptor 5C1.
OR5H2	Olfactory receptor 5H2.
OR6B2	Olfactory receptor family 6 subfamily B member 2.
OR8I2	Olfactory receptor 8I2.
PKP3	Plakophilin-3. May play a role in junctional plaques.
PLBD1	Phospholipase B-like 1. In view of the small size of the putative binding pocket, it has been proposed that it may act as an amidase or a peptidase (By similarity).
RBM34	RNA-binding protein 34.
RDX	RDX protein. May regulate binding of cytoskeletal proteins.
REXO1	RNA exonuclease 1 homolog. Seems to have no detectable effect on transcription elongation in vitro.
SIAE	Sialate O-acetylesterase. Catalyzes the removal of O-acetyl ester groups from position 9 of the parent sialic acid, N-acetylneuraminic acid.
SIMC1	SUMO-interacting motif-containing protein 1.
STK31	Serine/threonine-protein kinase 31.
TEX30	Testis-expressed protein 30. Has hydrolase activity.
THSD7B	Thrombospondin type-1 domain-containing protein 7B.
TMEM255A	Transmembrane protein 255A
TNXB	TNXB protein. May regulate collagen fibril organization.
TPTE	Putative tyrosine-protein phosphatase TPTE. Could be involved in signal transduction.
TRIM29	Tripartite motif-containing protein 29. It is able to complement the radiosensitivity defect of an ataxia telangiectasia (AT) fibroblast cell line.
TTC27	Tetratricopeptide repeat protein 27.
TTC29	Tetratricopeptide repeat protein 29.
UBN2	Ubinuclein-2.
WFDC10B	WAP four-disulfide core domain 10. Integral membrane component.
ZNF226	Zinc finger protein 226.
ZNF250	Zinc finger protein 250. May have transcription factor activity.
ZNF330	Zinc finger protein 330. Binds zinc and metal ions.
ZNF681	Zinc finger protein 681.
ZNF770	Zinc finger protein 770. Has RNA polymerase II transcription factor activity, sequence-specific DNA binding.
ZNF814	Putative uncharacterized zinc finger protein 814.
ZNF844	Zinc finger protein 844.
